# Autism spectrum disorder in architecture perspective: a review of the literature and bibliometric assessment of research indexed in Web of Science

**DOI:** 10.12688/f1000research.54437.1

**Published:** 2021-10-26

**Authors:** Reham Moniem Ali, Deema Faisal Al-Saleh, Khadeeja M N Ansari, Hala A. El-Wakeel, Mai Ibrahim Shukri

**Affiliations:** 1 Interior Design Department, College of Design, Imam Abdulrahman bin Faisal University, Saudi Arabia, Eastern Province, PO. 1982, Saudi Arabia

**Keywords:** Autism; Autism Spectrum disorder; Bibliometrics; Scientometric; Architecture; Relevant source, Web of science.

## Abstract

*Purpose:*

The primary objective of this research paper was to explore the current state-of-the-art research on autism spectrum disorder from a designer's perspective. An increasing number of scholarly publications in this discipline have urged researcher interest in this topic; however, there is still a lack of quantitative analysis. Therefore, this paper aims to analyze global research output on autism spectrum disorder from a designer's perspective during 1992–2021.

Methodology:

A bibliometric method was employed to analyze the published literature from 1992–2021. 812 papers were downloaded from the Web of Science core collection for analysis focused on annual growth of literature, prolific authors, authorship pattern, productive organizations, countries, international collaboration, literature trends by keyword analysis, and identifying the funding agencies. Various bibliometrics and scientometrics software were used to analyze the data, namely Bibexcel, Biblioshiny, and VOS viewer.

Results:

There were 812 research papers published in 405 sources during 1992–2021. 2019 was noted as the most productive year (NP=101), and 2014 received the highest number of citations (TC=6634). Researchers preferred to publish as journal articles (NP=538; TC=24922). The University of Toronto, Canada, was identified as a productive institution with 42 publications and 5358 citations. The USA was the leading producing country with 433 publications, and most of the researchers publish their work in the journal "Scientific Reports" (NP=16). The word "autism" (NP=257) and "architecture" (NP=165) were the most frequently used keywords in autism research.

## Introduction

Autism spectrum disorder (ASD) is a complicated neurological disorder that until now has been inscrutable. The population of individuals on the spectrum worldwide is increasing due to the increased awareness. As their numbers grow, professionals in many fields started studying their ASD cases to provide them with a better life ( Hauptman *et al.*, 2019). Individuals on the spectrum are part of a growing population usually ignored in design despite the current tendency to create designs that focus on persons with special needs. There are binding recommendations and laws on designing buildings that respect physical disabilities, and the field is rich in design applications for physical needs ( Sánchez *et al.*, 2011). By contrast, there is utter indifference towards the person with mental health disabilities. The built environment can cause extra confusion, which leads to a negative impact on children with ASD. Environmental and behavioral research has profoundly influenced the practice of interior architecture. Architects and interior architects are responsible for providing an inclusive built environment to improve the quality of life, especially for people with special needs ( Kopec, 2012).

A vast amount of literature has been published on autism in medical and psychological journals over the years. However, few studies from an architectural and interior architecture perspective have been published. Recently, architects have become interested in finding out the relationship between environment and autistic behavior to provide a suitable environment and support wellbeing. Today's literature is based on disability studies, environmental behavior studies, environmental design considerations, and guidelines to address behavioral aspects for autistic children. This study will cover this knowledge gap, and the literature review will progress from the general concept of autism to focus on autism and the physically built therapeutic environment.

## Literature review

ASD is a neurodevelopmental condition that affects children from a young age. It is marked by functional impairment in social communication, limited interests, and repetitive habits, as well as hypersensitivity to touch, vision, taste, or sound in certain people. Autistic disorder, high-functioning autism (HFA), Asperger syndrome (AS), pervasive developmental disorder-not otherwise specified (PDD-NOS), and atypical autism are all diagnostic terminology that has previously been employed. ASD is expected to affect one out of every 88 children in the United States, with one out of every 56 boys being affected. ( Taghizadeh *et al.*, 2015)

The diagnosis rates for ASD have increased sharply worldwide in the last 40 years compared with other disabilities. The environment plays a role in human behavior. ASD children have sensory processing difficulties, which create challenges in understanding the surrounding environment, thus affecting their behaviors negatively ( Sánchez *et al.*, 2011).

Pallasmaa (2005) diagnosed with ASD, said: 'I confront the city with my body.' The interaction between a person and their environment produces many physical and mental challenges for ASD. Therefore, the built environment is an important factor that significantly inﬂuences, directly and indirectly, individuals' behavior. ASD children are a special case, which should be defined to help them access space and inhabit it. Two issues must be considered to understand the impact of the environment on the development of one's life ( Horne, 1997):

1- The identification of the physical environment in its material and symbolic context.
2- The impact of the environment on one's behavior and how people perceive themselves and their surroundings.

Autistic people have difﬁculties in processing the information from the physical environment through their senses, and they are forced to exert more effort to understand it. The difficulty in understanding provokes frustration and erratic behavior.

A vast amount of literature has been published on autism in medical and psychological journals over the years. However, few studies from an architectural perspective have been published even though the role of the sensory environment in autistic behavior has been an issue of debate since Leo Kanner first defined the disorder in 1943 ( Kanner, 1943). Recently, architects have become interested in finding out about the relationship between environment and autistic behavior to provide a suitable environment and support wellbeing.

Few interior designers and architects have yet started to define codes and guidelines as a design solution for ASD to build autism-friendly surroundings that support users with ASD and prepares them to face other environments. The designer's approach is usually to compare between children with ASD and without through their behaviors to find the differences in their needs in the environment ( Delmolino & Harris, 2012). Environmental and behavioral research has profoundly influenced architecture, and there is a growing need and trend towards user-centered and evidence-based design research.

### Theoretical models of autism

Many human–environment interaction research conducted by environmental psychologists have focused on the environment's psychological factors rather than the physical setting. This section will clarify the relationship between autism and the environment.

**1- Human ecosystem (HES)**

In 1992, Guerin defined the Human ecosystem (HES) theory model in a learning environment to understanding autistic behavior. The variables in this progress are related to the specific model components:

a. HO, human organism: gender, age, number of children, and the level of diagnosing
b. DE, designed environment: control of entry and exit (safety/security); classroom configuration and adaptability to make changes; lighting (artificial light/daylight); acoustics/noise; thermal comfort (temperature, humidity, ventilation, *i.e.* indoor air quality); wayfinding; building; FF&E (furnishings, fixtures, and equipment) materials and finishes (color, pattern) ( Kopec, 2012; Martin & Guerin, 2010).
c. NE, natural environment: access to daylight and natural ventilation, as well as green space and/or water ( *i.e*. landscape elements).
d. SE, social environment: visual, auditory, and physical communication method, as well as communication and interaction among children and caregivers in the same physical area.

**2- Performance prediction model (PPM)**

The performance prediction model (PPM) describes the transactions between the users and their physical environment through the behavior. Also, understand how the physical environment affects user variables by observing behavior. In addition, clarify the interaction between the three components to lead to universal design principles. Even though this model is not explicitly created for ASD children, the research can be applied to users with different personal characteristics or functional abilities. This model consists of three main components (user variables, behavior, and environment). The variables in this progress are related to these specific components:

a. User abilities: individual characteristics and functional abilities.
b. Task outcome: behavior and experiential.
c. Physical environment: physical characteristics, organization, and ambience.
d. Universal design: equitable use, flexibility in use, simple and intuitive, perceptible information, tolerance for error, low physical effort and size and space for approach and use.

This model is used as a guide for the designer in designing different types of the physical environment for different users because it helps to categorize the users according to their characteristics, which are:

• Cognitive abilities: include all complex mental function prosses to make an action, for example, decision-making and planning ( ICF illustration library, 2021)
• Social and communication: include all components of the communication process with others by using different devices and methods to deliver or perceive massages ( World Health Organization, 2017)
• Sensory functions: includes touch, smell, visual, and hearing systems ( ICF illustration library, 2021)
• Mobility: the ability to manage body movements such as changing body position or location, carrying objects, performing physical activities ( ICF illustration library, 2021)

The characteristics of autism are varied in intensity, degree, and amount and manifest differently from person to person and over time. The common characteristics associated with ASD are loosely based on the DSM-5, common features of ASD, and PMM on ASD.

1. Cognitive abilities
2. Social and communication interaction
3. Sensory function
4. Activity performance

There is limited research on how environments may affect behavior and be designed to meet the needs of those with ASD. Also, there is a lack of information on the experience of spaces and perceptions by people with autism. This research will try to fill this missing gap and help with understanding the interaction between children with ASD and their physical environment.

**3- Theoretical underpinnings of design**

Interior designers concentrate on the design of the interior environment with the requirements of the person who will be inhabiting the space as the driving force behind all design decisions. Human factors, lighting, occupant wellbeing and performance, post-occupancy evaluation, research, theories about the relationship between human behavior and the designed environment, and universal design are among the ten knowledge areas covered by the 'Human Environment Needs: Research and Application' (HEN) category.

Experts on ASD consider that the first six years of school, from preschool to sixth grade, are important in reaching children and laying the groundwork for their lifelong learning and general well-being. Even when daily activities are meticulously organized, classrooms attended by children with ASD, or other children are highly dynamic, unpredictable environments. Because of this instability, examining the architecture of classroom space in schools where children with ASD attend from preschool to sixth grade is difficult. However, the framework identified by ( Guerin, 1992), which recognized the interaction of the human organism (HO), the BTE, the natural environment (NE), and the behavioral environment (BHE).

## Research questions

1) What are the annual research trends of autism in architecture during 1992–2021?
2) What are the most important types of research in autism?
3) Which authors are the most prolific, and what is the authorship trend in autism research?
4) What are the most relevant journals in journals in autism?
5) What are the most important organizations and counties in autism?
6) What are the most used keywords of autism in the field of architecture?
7) What are the most global collaborative countries producing scientific literature on autism?
8) What are the most cited documents and cited references in autism?
9) What the most influential funding agencies?

## Research methodology

Statistical techniques are used to analyze different types of publications such as books, conferences, journal articles, *etc*., known as bibliometrics. Scientometrics is the sub-field of bibliometrics that studies quantitative means of investigation, scholarly publishing practices, publishing trends, trend topics, *etc*. This study, therefore, applies the scientometric method to ASD in the architecture field. The required literature in autism retrieved from Web of Science (as of 4 ^th^ June 2021).

The following search query involved in the Web of Science database ( Clarivate Analytics, 2020)

• TOPIC: "autism"
• Refined by: TOPIC: "architecture"
• Further refined by language: English
• Timespan: All years. Indexes: SCI-EXPANDED, SSCI, A&HCI, CPCI-S, CPCI-SSH, ESCI.

**Figure 1.**
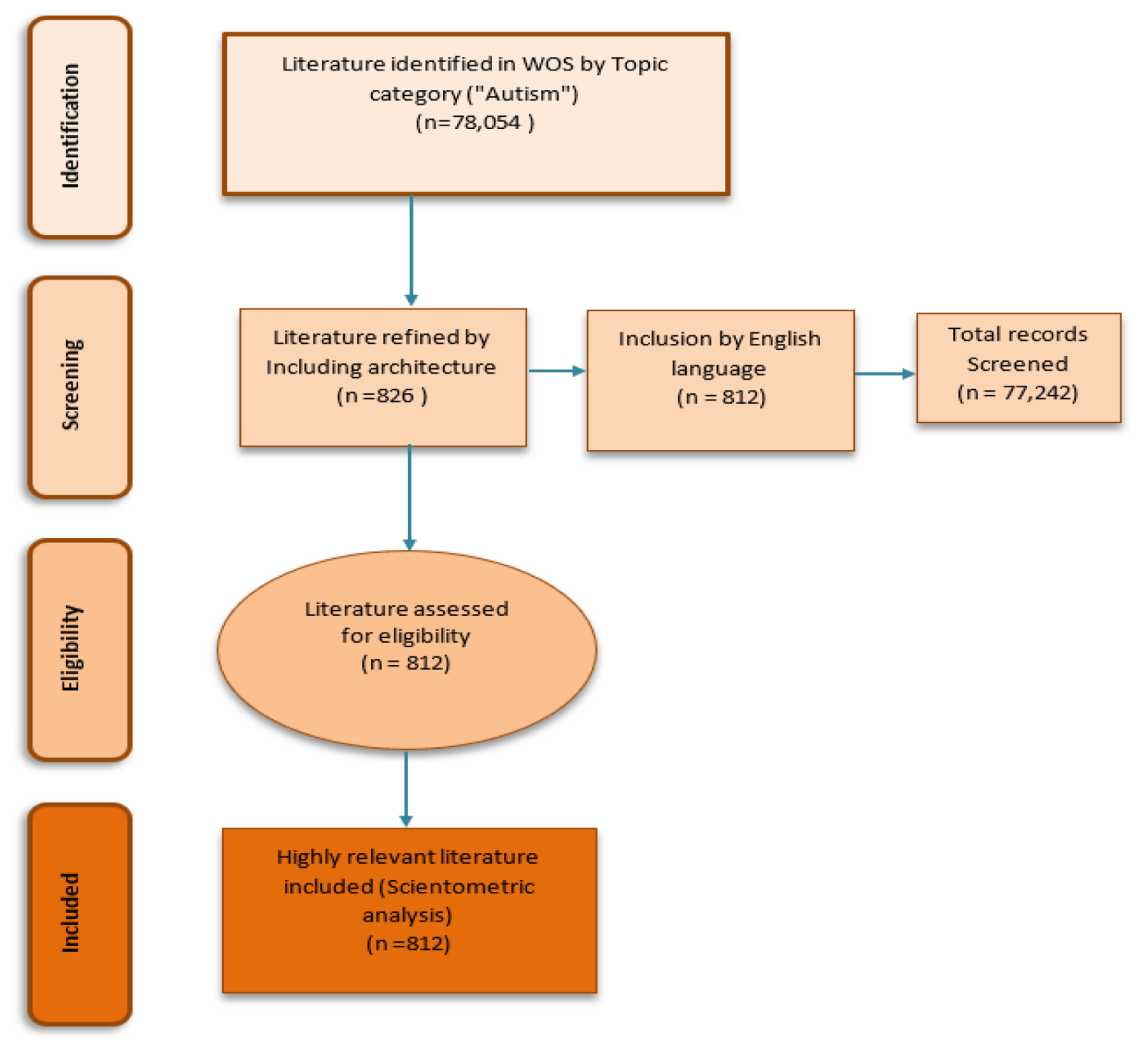
Four phase flow chart of data extraction and filtration process.

812 documents have been retrieved ( Figure 1) for final analysis during 1992–2021. All the research data was downloaded in BibTeX, Tab-Delimited (win), plain text, and analyzed with Microsoft Excel (RRID:SCR_016137; Google Sheets (RRID:SCR_017679) is an open access alternative) and Scientometric and bibliometrics tools, namely Bibexcel ( Persson *et al.*, 2009), Biblioshiny ( Aria & Cuccurullo, 2017), and VOSviewer ( van Eck & Waltman, 2010).

## Results and discussion

From 1992 to 2021, 405 sources were contributed by 5088 authors with 812 papers in autism. Single authored documents were 61 papers; hence authors in autism produce more research in collaboration. The average number of years of publications is 5.74, the average number of citations per document 43.21, and the average number of citations per year per document 5.711. 36,654 references have been consulted to produce 812 research papers. The number of documents per author is 0.16, authors per document are 6.27, Co-authors per document is 8.16, and the collaboration index is 6.71.

### Annual research growth and citation's structure in autism spectrum disorder during 1992–2021

The first research paper on autism was recorded in 1992 with 382 citations (no publication indexed in 1993, 1995, 1996, 1997, and 2003), similar results reported by ( Kumar *et al.*, 2021). Though the research output gradually increases, but shallow up until 2012. The autism research increased markedly after 2013, noticeably more than 50 papers appeared every year after 2013. The year 2019 was the most successful in term of the number of the article (NP=101), followed by the year 2016 and 2017, in which the second highest number of research papers published, coincidently the year 2018 and 2020 have equal number published articles (NP=84) and the year 2021 have 35 papers with 19 citations. The highest number of citations received in 2014 (TC=6634) for 53 publications, followed by the year 2011 (TC=4078) for 31 papers and the year 2010 (TC=3108, TP=34) ( Table 1).

**Table 1.** Annual research growth and citation's structure.

Year	NP	TC	Citation sum within h-core	h-index
**1992**	1	382	382	1
**1994**	1	0	0	0
**1998**	2	29	29	2
**1999**	4	235	235	3
**2000**	3	174	174	3
**2001**	1	0	0	0
**2002**	2	126	126	2
**2004**	5	136	136	4
**2005**	3	330	329	2
**2006**	7	557	553	6
**2007**	11	2196	2188	10
**2008**	18	628	614	13
**2009**	22	1547	1522	16
**2010**	34	3108	3000	26
**2011**	31	4078	4008	24
**2012**	31	2360	2301	24
**2013**	51	2467	2239	28
**2014**	53	6634	6305	30
**2015**	58	2093	1818	22
**2016**	85	2549	1972	27
**2017**	85	1965	1396	23
**2018**	84	1726	1240	21
**2019**	101	1451	1035	16
**2020**	84	288	139	10
**2021**	35	19	13	3

*NP=Number of Publication **TC=Total Number of Citations

### Type of research papers

The journal articles (NP=537) were the most preferred form, which agrees with ( Rahaman *et al.*, 2021b). The review found a second preferred form (NP=142), followed by proceedings papers (NP=71) and then meeting abstract (NP=17). Other documents were minor in the list, published only three papers each. On the other hand, the articles also received the highest number of total cations (24922), followed by review (TC=8916) ( Table 2).

**Table 2.** Type of research.

Rank	Document type	NP	TC	Citation sum within h-core	h-index
**1**	Article	538	24922	16725	73
**2**	Review	142	8916	7085	45
**3**	Proceeding’s paper	71	183	92	7
**4**	Meeting abstract	17	0	0	0
**5**	Article Proceedings paper	12	436	419	7
**6**	Review; book chapter	8	383	381	5
**7**	Editorial material	7	162	160	4
**8**	Article; early access	7	11	9	2
**9**	Review; early access	4	1	1	1
**10**	Book chapter	3	55	54	2
**11**	Letter	3	21	21	2

*NP=Number of Publication **TC=Total Number of Citations

### Productive organization

It is evident that the top ten organizational productivity ranges between 25 to 42 publications ( Table 3). The University of Toronto is the leading organization in autism research (NP=42), followed by Vanderbilt University (NP=37), University of California, Los Angeles (NP=35), Yale University (NP=33), and Massachusetts General Hospital (NP=30). Harvard Medical School (NP=25) identified as the minor producer of research in the top ten list. Interestingly, most of the listed organization are in the USA (9 organizations), and one organization from Canada. Stanford University was the most cited organization (TC=6686) for 28 publications, followed by Yale University (TC=6059) for 33 research in autism.

**Table 3.** Top ten organization-wise research in autism.

Rank	Affiliation	Country	NP	TC	Citation sum within h-core	h-index
**1**	Univ Toronto	Canada	42	5358	5162	22
**2**	Vanderbilt Univ	USA	37	5529	5407	27
**3**	Univ Calif Los Angeles	USA	35	5302	5184	23
**4**	Yale Univ	USA	33	6059	5987	22
**5**	Massachusetts Gen Hosp	USA	30	4707	4611	20
**6**	Univ Calif San Francisco	USA	30	4499	4449	17
**7**	Stanford Univ	USA	28	6686	6643	19
**8**	Univ Calif San Diego	USA	27	2541	2476	17
**9**	Hosp Sick Children	Canada	26	4201	4132	18
**10**	Harvard Med Sch	USA	25	1013	950	14

### Productive country

Moreover, it is found that the top eight countries produced over 50 research papers ( Table 4). Only two countries have over 100 articles on autism. The USA had outstanding research output in autism with 433 publications and 27124 citations, followed by the UK (118 publications, 7569 citations), Canada (79 publications, 6816 citations), China (72 publications, 3339 citations), and France (60 publications, 3304 citations). The analyses reveal that half of the research in autism contributed by the USA that received the highest number of citations (TC=27124) for 433 publications, followed by the UK with 7569 citations with 118 publications, and Canada with 6816 citations and 79 publications. Australia managed minimum citation (TC=2048) in the list with 46 publications.

**Table 4.** Top ten country-wise research in autism.

Rank	Country	NP	TC	Citation sum within h-core	h-index
**1**	USA	433	27124	19409	76
**2**	UK	118	7569	6612	37
**3**	Canada	79	6816	6281	31
**4**	Peoples R China	72	3339	2970	20
**5**	France	60	3304	3027	22
**6**	Germany	59	5706	5424	24
**7**	Italy	59	3263	2938	21
**8**	Netherlands	55	4490	4213	26
**9**	Australia	46	2048	1856	20
**10**	Sweden	36	4499	4368	20

### The relevant sources in ASD

All the top ten sources have more than 12 publications; coincidentally, six sources ( *American Journal of Human Genetics, American Journal of Medical Genetics Part B-Neuropsychiatric Genetics, Biological Psychiatry, Molecular Autism, Molecular Psychiatry, Neuron*) produced 12 publications each. *Scientific Reports* (Nature Publishing Group) was considered the most relevant source with 14 publications and 203 citations, followed by *Nature Neuroscience* (Nature Publishing Group) with 14 publications and 1986 citations and *Human Molecular Genetics* and *Plos One* with 13 publications each and 1015 and 371 citations, respectively. The analysis reveals that most of the sources belongs to the Q1 category (eight sources), and two in Q2 category. The highest impact factor journal in the list was *Nature Neuroscience* (JIF=20.07), followed by *Neuron* (JIF=14.41) and *Molecular Psychiatry* (JIF=12.38) ( Table 5).

**Table 5.** Top ten appropriate sources in autism.

Rank	Source	JIF	Q	Country	Publisher	NP	TC	h_index	g_index	m_index
**1**	*Scientific Reports*	3.99	Q1	UK	Nature	16	203	9	14	1.29
**2**	*Nature Neuroscience*	20.07	Q1	USA	Nature	14	1986	11	14	1.00
**3**	*Human Molecular Genetics*	5.1	Q1	UK	Oxford University Press	13	1015	10	13	0.83
**4**	*Plos One*	2.74	Q2	USA	Public Library of Science	13	371	9	13	0.75
**5**	*American Journal of Human Genetics*	10.5	Q1	USA	Cell Press	12	1093	10	12	0.67
**6**	*American Journal of Medical Genetics* *Part B-Neuropsychiatric Genetics*	3.38	Q2	USA	Wiley-Liss	12	293	8	12	0.67
**7**	*Biological Psychiatry*	12.09	Q1	USA	Elsevier	12	439	10	12	0.77
**8**	*Molecular Autism*	5.86	Q1	USA	BMC	12	226	9	12	0.82
**9**	*Molecular Psychiatry*	12.38	Q1	USA	Nature	12	1290	9	12	
**10**	*Neuron*	14.41	Q1	USA	Cell Press	12	1612	11	12	0.73

*NP=Number of Publication **TC=Total Number of Citations ***JIF=Journal impact factor ****Q=Quartile

Interestingly, we found that of 347 sources identified in autism spectrum research, only two were from the field of architecture, namely *Archnet-Ijar International Journal of Architectural Research* (NP=3, TC=5) and *Architectural Design* (NP=1, TC=2).

### Prolific authors

This analysis reveals that the article range of authors varied between nine and 12. Five authors (Devlin B, Geschwind DH, Scherer SW, State MW, and Wang Y) emerged as the most prolific authors with 13 publications each, 4383, 3409, 3338, 3662, and 333 citations, respectively. Buxbaum JD (Icahn School of Medicine at Mount Sinai) found as the second highest prolific author with 13 publications and 2970 citations, followed by Bourgeron T, Eichler EE, and Li Y with 11 publications and 2142, 1944, and 568 citations, respectively. Casanova MF (University of South Carolina School of Medicine) noted as the least contributed authors in the top ten list with nine publications and 361 citations. Devlin B (Mount Sinai School of Medicine) was the most cited author with 4383 citations for 13 publications, followed by Geschwind DH with 3409 citations for 13 publications, and Wang Y (Carnegie Mellon University) managed only 333 citations for 13 publications. The table also shows that the most prolific authors belong from the USA (7 authors), followed by Canada, France, and China. ( Table 6).

**Table 6.** Top ten most productive authors in autism research.

Rank	Author	Affiliation	Country	NP	TC	h_index	g_index	m_index
**1**	Devlin B	Mount Sinai School of Medicine	USA	13	4383	12	13	0.80
**2**	Geschwind DH	University of California	USA	13	3409	12	13	0.80
**3**	Scherer SW	University of Toronto	Canada	13	3338	12	13	0.80
**4**	State MW	Mount Sinai School of Medicine	USA	13	3662	12	13	0.86
**5**	Wang Y	Carnegie Mellon University	USA	13	333	9	13	1.00
**6**	Buxbaum JD	Icahn School of Medicine at Mount Sinai	USA	12	2970	10	12	0.63
**7**	Bourgeron T	Université de Paris	France	11	2142	9	11	0.60
**8**	Eichler EE	University of Washington,	USA	11	1944	9	11	0.75
**9**	Li Y	Peking University	China	11	568	7	11	1.17
**10**	Casanova MF	University of South Carolina School of Medicine	USA	9	361	9	9	0.45

*NP=Number of Publication **TC=Total Number of Citations

### The pattern of authorship

**Figure 2.**
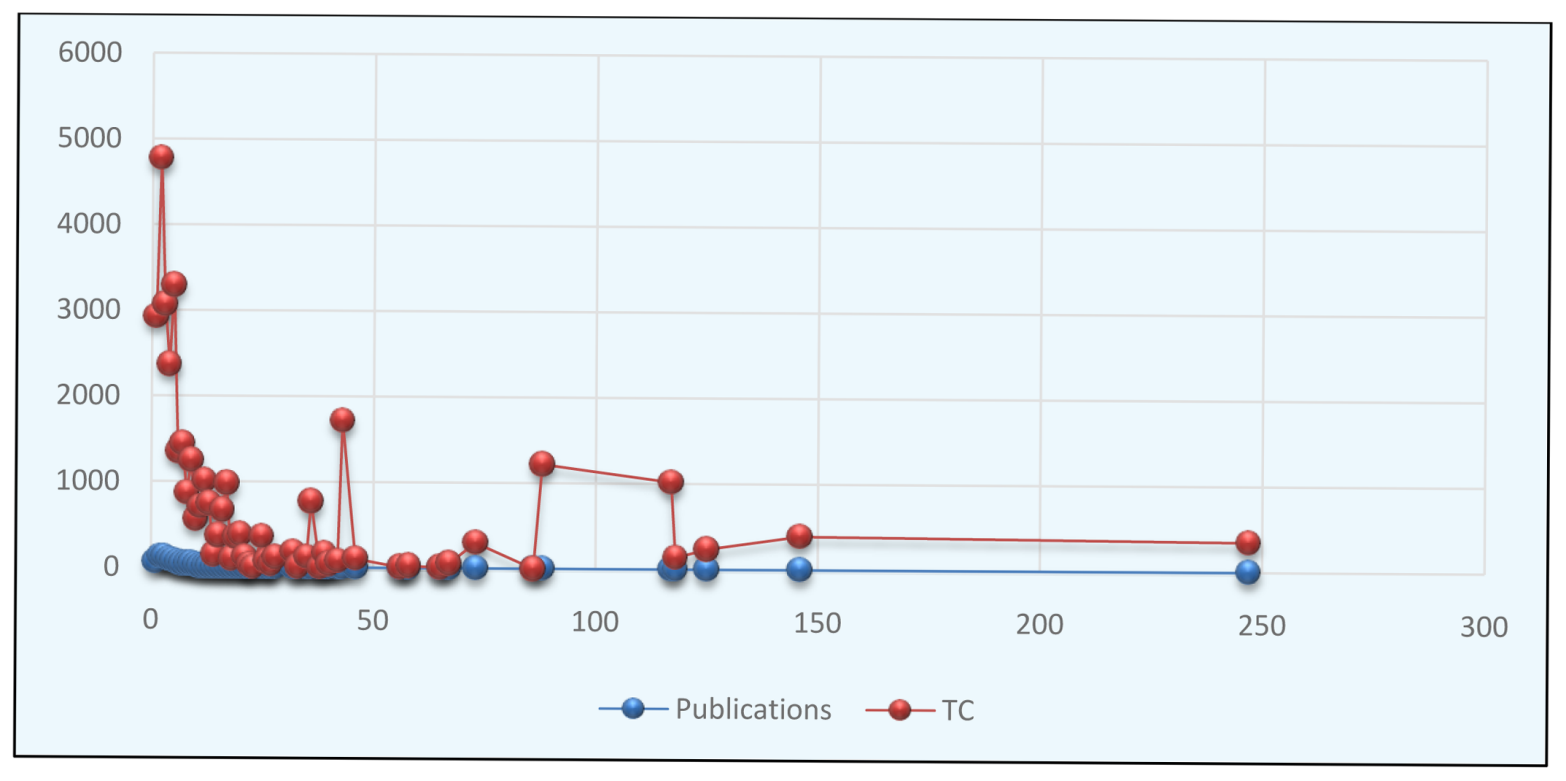
Pattern of authorship in autism.

The Figure 2 illustrated the pattern of authorship in autism literature. It was clear from the figure that the authorship pattern ranged from single to two hundred and forty-seven. The analysis reveals that collaborative research is more prominent among the research of autism over the study period. The top six authorship patterns produced over 50 publications in the field. Three authorship patterns (NP=123) contributed a maximum article in autism, followed by two authorship (NP=120), four authorship (NP=93), five authorship (NP=79), single authorship (NP=61), and six authorship (NP=56). The authorship of 27, 36, 38, 39, 40, 42, 46, 56, 58, 65, 67, 73, 86, 88, 118, 125, 146, and 247 each contributed only single publications in autism. The results also showed that two authorship patterns received the highest number of citations (TC=4775), followed by five authorship (TC=3296) and Three authorship (TC=3071). Rahaman conducted a similar type of authorship pattern analysis ( Rahaman *et al.*, 2021a).

### Mapping co-occurrence of all keywords (author and indexed)

**Figure 3.**
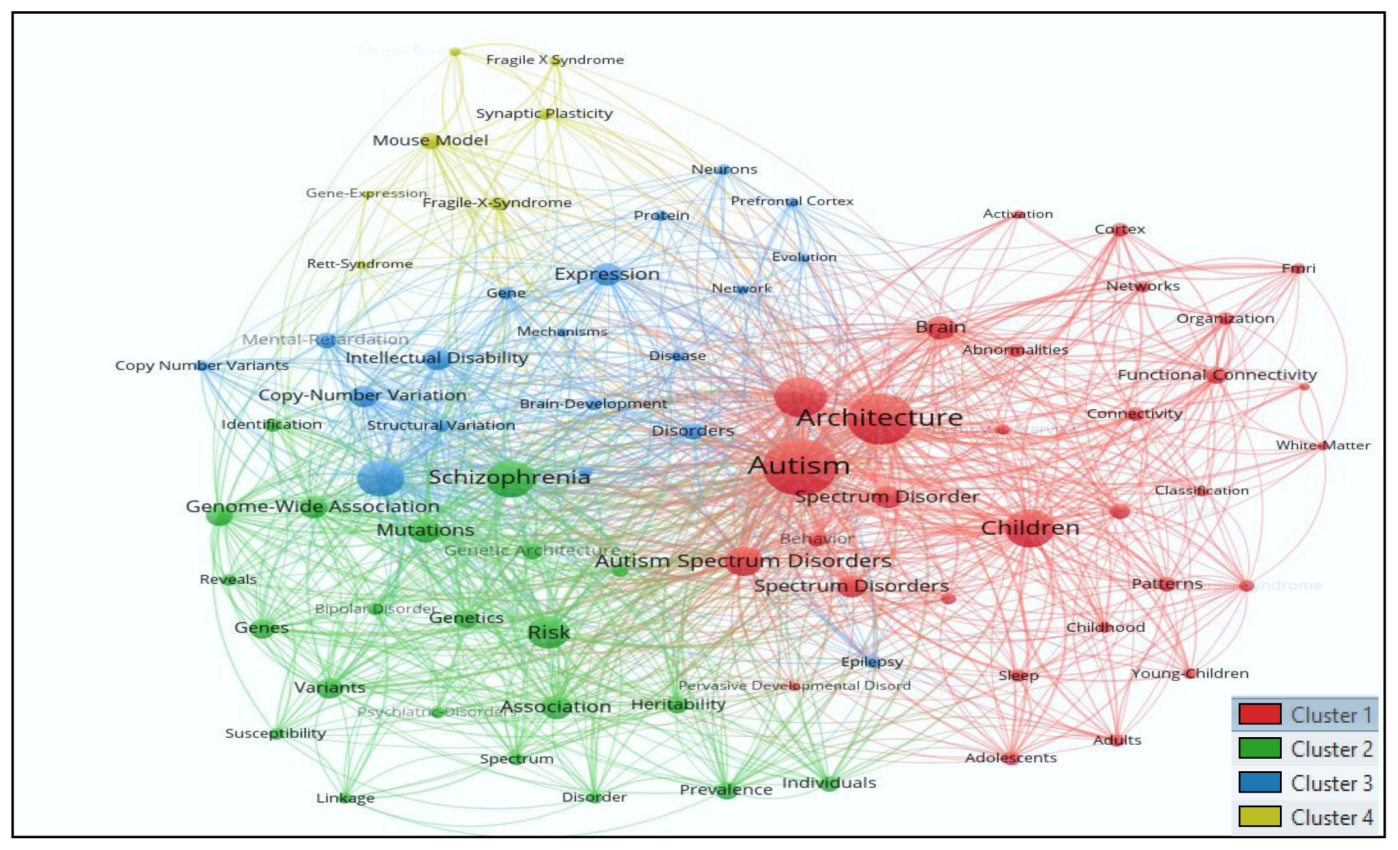
Keyword analysis using Vosviewer.

**Figure 4.**
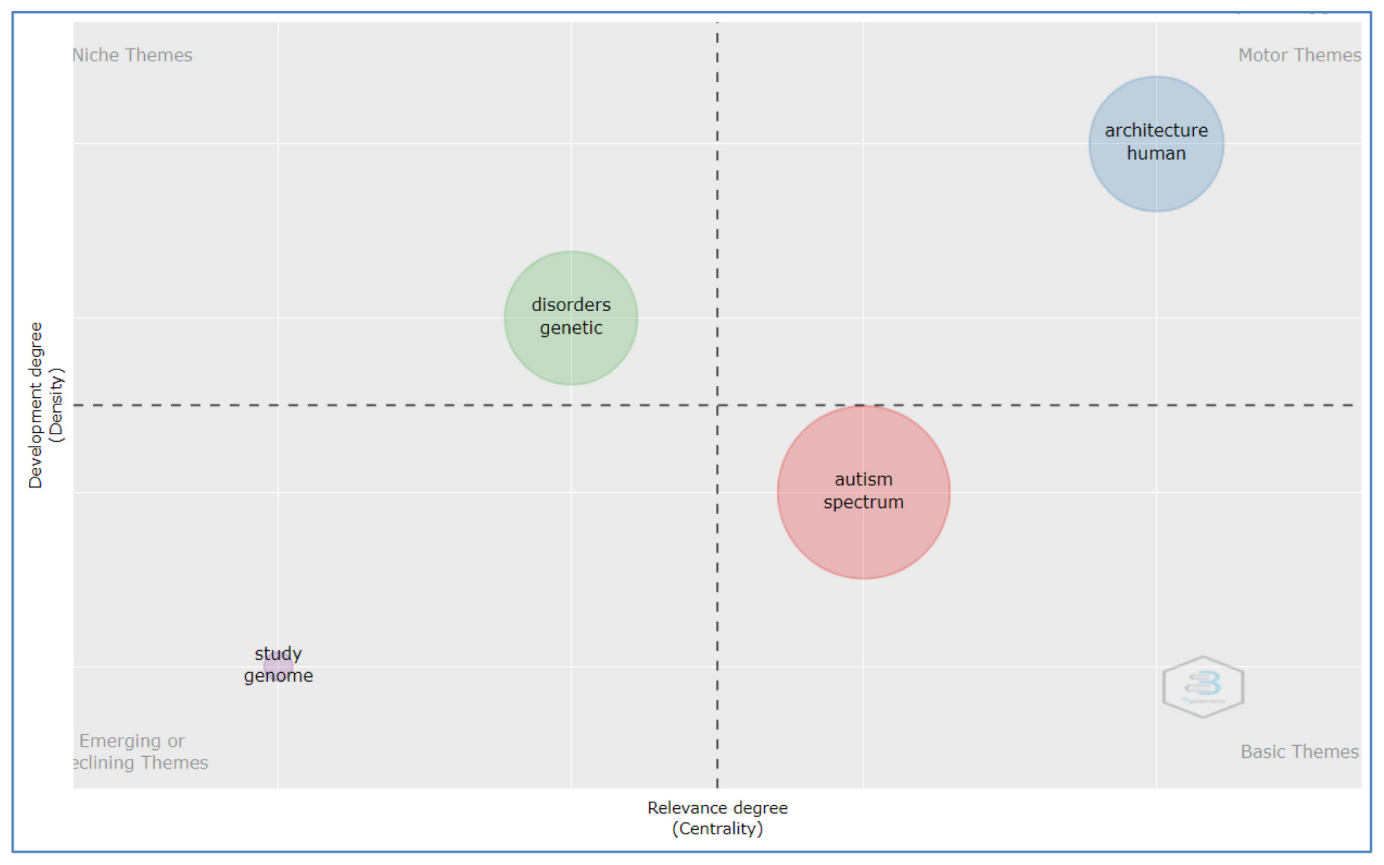
Thematic map by title analysis.

Figure 3 shows analysis of all keywords that have been used in autism research during 1992–2021. The results showed that 3848 keywords appeared in autism research. To map the co-occurrence of all the keywords, minimum of 15 occurrences of keywords were considered for analysis. Out of 3848 keywords, only 79 keywords met the thresholds, and all 79 selected keywords are clustered in Figure 4 with 1737 links and total link strength (5557). The size of the ball indicates a strong network of keywords, with each color representing a distinct cluster.

Cluster *1* comprises 31 keywords (abnormalities, activation, adolescents, adults, architecture Asperger-syndrome, autism, autism spectrum disorder, autism spectrum disorders, behavior, brain, childhood, children, classification, connectivity, cortex, diagnostic interview, fMRI, functional connectivity, high-functioning autism, human cerebral-cortex, meta-analysis, networks, organization, patterns, pervasive developmental disorders, sleep, spectrum disorder, spectrum disorders, white-matter, and young-children).

Cluster *2* has 22 keywords (association, bipolar disorder, copy number variation, disorder, genes, genetic architecture, genetics, genome-wide association, heritability, identification, individuals, linkage, mutations, phenotype, prevalence, psychiatric-disorders, reveals, risk, schizophrenia, spectrum, susceptibility, and variants).

Cluster *3* includes 19 keywords (brain-development, copy number variants, copy-number variation, de-novo mutations, disease, disorders, epilepsy, evolution, expression, gene, intellectual disability, mechanisms, mental-retardation, network, neurodevelopmental disorders, neurons, prefrontal cortex, protein, and structural variation).

Cluster *4* has seven keywords (fragile x syndrome, fragile-x-syndrome, gene-expression, mental-retardation protein, mouse model, rett-syndrome, and synaptic plasticity).

The top ten keywords were autism (frequency=257), architecture (165), autism spectrum disorder (127), children (123), schizophrenia (92), autism spectrum disorders (91), de-novo mutations (86), Risk (73), brain (59) and expression (freq.=55) had weighty number of occurrence with strong total link strength.

### Thematic map by title

Figure 4 shows four alternative typologies of themes that can be visualized using a thematic map. The thematic parameter considered as, title selected for field, minimum number of words selected 80 and Unigram selected for graph.

The basic theme: Autism spectrum which represented by cluster *1* (autism, spectrum, disorder, children, brain, network, functional, connectivity, based, analysis, sleep, neural, developmental, learning, networks, structural, reveals, system, approach, design, matter, review, robot, resting, control, developing and white).

The motor theme: architecture human in cluster *2* (architecture, human, gene, syndrome, social, development, cortical, protein, autistic, model, synaptic, fragile, neuronal, cognitive, ASD, altered, behavior, mental, mice, role, cortex, expression, function, visual, cell, mouse, processing, and activity.

Niche theme: genetic disorder placed in cluster *3* (disorders, genetic, variants, risk, schizophrenia, neurodevelopmental, genes, psychiatric, rare, common, de, genetics, novo, genomic, related, mutations, copy, disease, mechanisms, and sequencing).

Emerging or declining theme: study genome represented by cluster *4* (study, genome, association, wide and evidence).

### Most cited research papers in autism

The top ten papers ( Table 7) have more than 300 citations, published between 2007 and2015. "Large-scale brain networks and psychopathology: a unifying triple network model" (2011) by Menon V, published in *Trends Cogn Sci* was the topmost cited paper (1425 citations) ( Menon, 2011), followed by "Synaptic, transcriptional and chromatin genes disrupted in autism" (2014) by De Rubeis S, appeared in " *Nature*" (1220 citations) ( De Rubeis *et al.*, 2014), "The contribution of de novo coding mutations to autism spectrum disorder" (2014) by Iossifov I, published in *Nature* (1118 citations) ( Iossifov *et al.*, 2014), "Mapping autism risk loci using genetic linkage and chromosomal rearrangements" (2007) by Szatmari (999 citations) ( Szatmari *et al.*, 2007). "Dendritic spine pathology in neuropsychiatric disorders" (2011) by Penzes (838 citations) ( Penzes *et al.*, 2011), and "A genome-wide scan for common alleles affecting risk for autism" was the least cited paper among the top ten (393 citations) ( Anney *et al.*, 2010). It was noticeable that half of the top ten cited papers were published by Nature Publishing Group. The article entitled "Synaptic, transcriptional and chromatin genes disrupted in autism" ( De Rubeis *et al.*, 2014) has the highest total citations per year (152.50).

**Table 7.** Top ten cited papers.

Rank	Title	Author	Yar	Source	TC	TC/Year	N/TC
**1**	Large-scale brain networks and psychopathology: a unifying triple network model ( Menon, 2011)	Menon V	2011	Trends Cogn Sci	1425	129.55	10.83
**2**	Synaptic, transcriptional and chromatin genes disrupted in autism ( De Rubeis *et al*., 2014)	De Rubeis S	2014	Nature	1220	152.50	9.75
**3**	The contribution of de novo coding mutations to autism spectrum disorder ( Iossifov *et al*., 2014)	Iossifov I	2014	Nature	1118	139.75	8.93
**4**	Mapping autism risk loci using genetic linkage and chromosomal rearrangements ( Szatmari *et al*., 2007)	Szatmari P	2007	Nature Genet	999	66.60	5.00
**5**	Dendritic spine pathology in neuropsychiatric disorders ( Penzes *et al*., 2011)	Penzes	2011	Nat Neurosci	838	76.18	6.37
**6**	The autism brain imaging data exchange: towards a large-scale evaluation of the intrinsic brain architecture in autism ( Di Martino *et al*., 2014)	Di Martino A	2014	Mol Psychiatr	769	96.13	6.14
**7**	Insights into Autism Spectrum Disorder Genomic Architecture and Biology from 71 Risk Loci ( Sanders *et al*., 2015)	Sanders Sj	2015	Neuron	563	80.43	15.60
**8**	Most genetic risk for autism resides with common variation ( Gaugler *et al*., 2014)	Gaugler T	2014	Nature Genet	542	67.75	4.33
**9**	Mapping Early Brain Development in Autism ( Courchesne *et al*., 2007)	Courchesne E	2007	Neuron	485	32.33	2.43
**10**	A genome-wide scan for common alleles affecting risk for autism ( Anney *et al*., 2010)	Anney R	2010	Hum Mol Genet	393	32.75	4.30

*N/TC=Normalized total citation

### Most Cited references in autism research

Table 8 explained the most top ten cited references in autism research. It is clear from the table that all listed references received more than 50 citations. Article entitled "Insights into Autism Spectrum Disorder Genomic Architecture and Biology from 71 Risk Loci" (2015) by Sanders SJ, appeared in 'Neuron' was the most cited ( TC=92) reference in autism research ( Sanders *et al.*, 2015), followed by an article named 'Synaptic, transcriptional and chromatin genes disrupted in autism (2014) by De Rubeis S with 91 citations ( De Rubeis *et al.*, 2014), 'and 'The contribution of de novo coding mutations to autism spectrum disorder' (2014) by Lossifov I with 91 citations and appeared in the journal Nature ( Iossifov *et al.*, 2014). The cited references 'De novo gene disruptions in children on the autistic spectrum (2012) by Iossifov I published in 'NEURON' was the most diminutive receiver of citation with 61 TC ( Iossifov *et al.*, 2012).

**Table 8.** top ten cited references.

Rank	Title	Author	Year	Source	TC
**1**	Insights into Autism Spectrum Disorder Genomic Architecture and Biology from 71 Risk Loci ( Sanders *et al*., 2015)	Sanders SJ	2015	NEURON	92
**2**	Synaptic, transcriptional and chromatin genes disrupted in autism ( De Rubeis *et al*., 2014)	De Rubeis S	2014	Nature	91
**3**	The contribution of de novo coding mutations to autism spectrum disorder ( Iossifov *et al*., 2014)	Iossifov I	2014	Nature	91
**4**	Strong association of de novo copy number mutations with autism ( Sebat *et al*., 2007)	Sebat J	2007	Science	79
**5**	Functional impact of global rare copy number variation in autism spectrum disorders ( Pinto *et al*., 2010)	Pinto D	2010	Nature	77
**6**	De novo mutations revealed by whole-exome sequencing are strongly associated with autism ( Sanders *et al*., 2012)	Sanders SJ	2012	Nature	73
**7**	Sporadic autism exomes reveal a highly interconnected protein network of de novo mutations ( O'Roak *et al*., 2012)	O'roak BJ	2012	Nature	70
**8**	Structural variation of chromosomes in autism spectrum disorder ( Marshall *et al*., 2008)	Marshall CR	2008	AM J HUM GENET	69
**9**	Patterns and rates of exonic de novo mutations in autism spectrum disorders ( Neale *et al*., 2012)	Neale BM	2012	Nature	68
**10**	De novo gene disruptions in children on the autistic spectrum ( Iossifov *et al*., 2012)	Iossifov I	2012	NEURON	61

### Highly influential funding agencies

There are only four funding agencies from the top 10 list which funded more than 100 research papers ( Table 9). National Institutes of Health renowned as leading funding agency (313 publications, 23087 citations), followed by the United States Department of Human Health Services (313 publications, 22759 citations), the National Institute of Mental Health (182 publications, 16164 citations), European Commission (111 publications, 8476 citations), and National Institute of Child Health Human Development (66 publications, 7927 citations). The Wellcome Trust appeared as the least influential funding agency among the top ten (36 publications, 3959 citations). The USA was dominant in the top ten list (six funding agencies), followed by the UK (three funding agencies) and one agency from the EU.

**Table 9.** Top ten funding agencies in autism.

Rank	Funding agencies	Country	NP	% Of 812	TC
**1**	National Institutes of Health	USA	313	38.547	23087
**2**	United States Department of Health Human Services	USA	313	38.547	22759
**3**	National Institute of Mental Health	USA	182	22.414	16164
**4**	European Commission	EU	111	13.67	8476
**5**	National Institute of Child Health Human Development	USA	66	8.128	7927
**6**	National Institute of Neurological Disorders Stroke	USA	62	7.635	7101
**7**	National Institute of General Medical Sciences	USA	50	6.158	2827
**8**	UK Research Innovation	UK	50	6.158	4947
**9**	Medical Research Council UK	UK	48	5.911	5097
**10**	Wellcome Trust	UK	36	4.433	3959

### Country collaboration in autism

**Figure 5.**
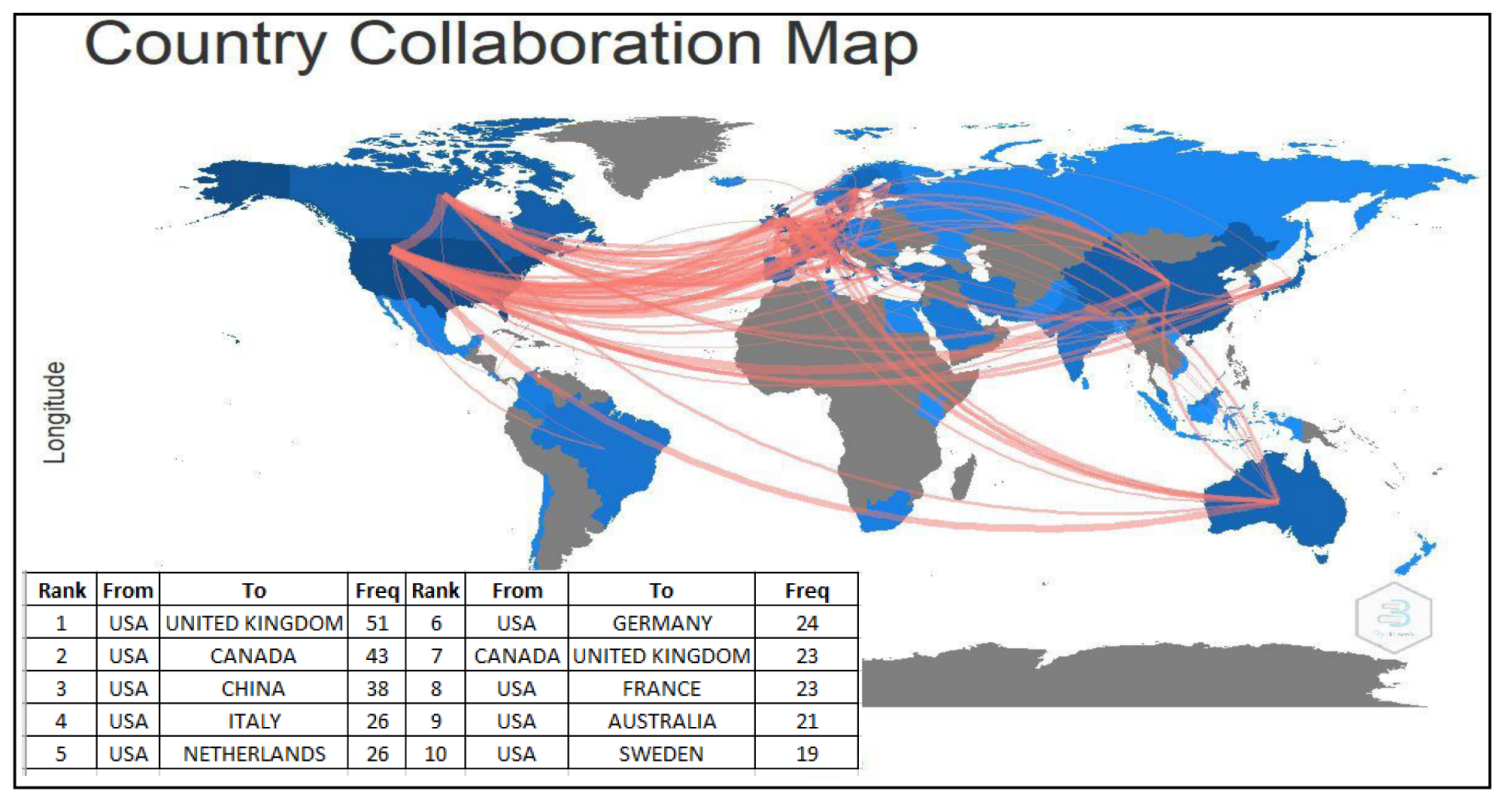
International collaboration map.

The most dominant country collaborations were the USA and United Kingdom (51 publications), followed by the USA and Canada (43 publications), the USA and China (38 publications), the USA and Italy (26 publications), and the USA and the Netherlands (26 publications). The USA with Sweden collaboration (19 publications) was listed at the bottom of the top ten list. It was interesting to show that the USA collaborated with nine countries (the UK, Canada, China, Italy, the Netherlands, Germany, France, Australia, and Sweden). The UK followed this with two countries (the USA and Canada). ( Figure 5).

## Conclusion

The purpose of this study considered to completely overview the published literature on ASD in the architecture field. However, no other bibliometric analysis has been done from 1992 to 2021 that comprehensively evaluates and summarizes the literature, progress, and future directions of this key sub-area of ASD. Moreover, researchers investigating ASD can use the findings of this study to build techniques that are specific to the themes that are currently being researched (by consulting the visualization of analysis of this study). They can also discover the most influential publications, authors, and journals in this field to uncover research gaps and fresh discoveries. Here are few noteworthy predictions and emerging trends (from this study in terms of ASD in architecture research) and future insights. In conclusion, this study contributed to identify that there is very limited research has been done in ASD in field of architecture, the researchers should focus and consider this area of research. The results also direct us to conduct more academic research in this area.

## Data availability

### Underlying data

Zenodo: Underlying data for 'autism spectrum disorder in architecture perspective: A review of the literature and bibliometric assessment of research indexed in Web of Science'. https://doi.org/10.5281/zenodo.5080242

Data are available under the terms of the Creative Commons Attribution 4.0 International license (CC-BY 4.0).
