# Autism spectrum disorder in architecture perspective: a review of the literature and bibliometric assessment of research indexed in Web of Science

**DOI:** 10.12688/f1000research.54437.2

**Published:** 2022-07-25

**Authors:** Reham Moniem Ali, Hala A. El-Wakeel, Deema Faisal Al-Saleh, Mai Ibrahim Shukri, Khadeeja M N Ansari

**Affiliations:** 1 Interior Design Department, College of Design, Imam Abdulrahman bin Faisal University, Saudi Arabia, Eastern Province, PO. 1982, Saudi Arabia

**Keywords:** Autism; Autism Spectrum disorder; Bibliometrics; Scientometric; Architecture; Relevant source, Web of science.

## Abstract

Purpose:

An increasing number of scholarly publications on autism spectrum disorder (ASD) have urged researcher interest in this topic; however, there is still a lack of quantitative analysis. Therefore, this study aims to cover the knowledge gap between the amount of literature published on ASD research on architectural and designers' perspectives compared to the medical and psychological fields. The study has analyzed global research output on ASD from a designer's perspective to recognize this gap related to designing the physical environment.

Methodology:

The bibliometric method was employed to analyze the published literature from 1992–to 2021. 812 papers were downloaded from the Web of Science for analysis based on annual growth of literature, prolific authors, authorship pattern, organizations, countries, international collaboration, and subject development by keywords and thematic map analyses. Various bibliometric and scientometric software was used to analyze the data, namely Bibexcel, Biblioshiny, and VOS viewer.

Results:

The812 research papers were published in 405 sources. 2019 appeared as a productive year (NP=101), and 2014 received the highest number of citations (TC=6634). Researchers preferred to publish as journal articles (NP=538; TC=24922). The University of Toronto, Canada, was identified as a productive institution with 42 publications and 5358 citations. The USA was the leading producing country with 433 publications, and most of the researchers published in the journal " *Scientific Reports*" (NP=16). The word autism (NP=257) and architecture (NP=165) were more frequently used keywords.

Conclusion:

The study identified a massive gap in the development of literature in ASD for architecture design and built environment perspective, the most important and trending keywords are missing, and the analyses also showed a lack of subject development. The authors have suggested areas and keywords for further research to fulfill the gap in the future.

## Background study of ASD

ASD is a neurodevelopmental condition that affects children from a young age. It is marked by functional impairment in social communication, limited interests, selective attention, repetitive habits, as well as hypersensitivity to touch, vision, taste, or sound in certain people ( Remington *et al.*, 2009). Autistic disorder, high-functioning autism (HFA), Asperger syndrome (AS), pervasive developmental disorder-not otherwise specified (PDD-NOS), and atypical autism is all diagnostic terminology that has previously been employed. ASD is expected to affect one out of every 88 children in the United States, with one out of every 56 boys being affected. ( Taghizadeh *et al.*, 2015). The diagnosis rates for ASD have increased sharply worldwide in the last 40 years compared with other disabilities ( Centers for Disease Control and Prevention, 2022)

Pallasmaa (2005), diagnosed with ASD, said: 'I confront the city with my body.' The interaction between a person and their environment produces many physical and mental challenges for ASD. Therefore, the built environment is an important factor that significantly influences individuals' behavior directly and indirectly. ASD children are a special case, which should be defined to help them access space and inhabit it. Two issues must be considered to understand the impact of the environment on the development of one's life ( Horne, 1997):

**1-**  The identification of the physical environment in its material and symbolic context.
**2-**  The impact of the environment on one's behavior and how people perceive themselves and their surroundings.

Autistic people have difficulties in processing the information from the physical environment through their senses, especially the influence of environmental stressors like noise and clutter, and they are forced to exert more effort to understand it. The difficulty in understanding provokes frustration and erratic behavior.

### Theoretical models of autism

Many human-environment interaction research conducted by environmental psychologists have focused on the environment's psychological factors rather than the physical setting. This section will clarify the relationship between autism and the environment.

**1- Human ecosystem (HES)**

In 1992, Guerin defined the Human ecosystem (HES) theory model in a learning environment to understand autistic behavior. The variables in this progress are related to the specific model components:

**a.**  HO, human organism: gender, age, number of children, and the level of diagnosing
**b.**  DE, designed environment: control of entry and exit (safety/security); classroom configuration and adaptability to make changes; lighting (artificial light/daylight); acoustics/noise; thermal comfort (temperature, humidity, ventilation, *i.e.* indoor air quality); wayfinding; building; FF&E (furnishings, fixtures, and equipment) materials and finishes (color, pattern) ( Kopec, 2012; Martin & Guerin, 2010).
**c.**  NE, natural environment: access to daylight and natural ventilation, as well as green space and/or water ( *i.e*. landscape elements).
**d.**  SE, social environment: visual, auditory, and physical communication method, as well as communication and interaction among children and caregivers in the same physical area.

Some researchers regarding the Nature of autism are convinced that autism is a pandemic of modern culture, with environmental factors at the roots such as pollution; researchers found early-life exposure to air pollution may be a risk factor for autism. ( Naviaux, 2012).

**2- Performance prediction model (PPM)**

The performance prediction model (PPM) describes the transactions between the users and their physical environment through the behavior. Also, understand how the physical environment affects user variables by observing behavior. In addition, clarify the interaction between the three components to lead to universal design principles. Even though this model is not explicitly created for ASD children, the research can be applied to users with different personal characteristics or functional abilities. This model consists of three main components (user variables, behavior, and environment). The variables in this progress are related to these specific components:

**a.**  User abilities: individual characteristics and functional abilities.
**b.**  Task outcome: behavior and experiential.
**c.**  Physical environment: physical characteristics, organization, and ambiance.
**d.**  Universal design: equitable use, flexibility in use, simple and intuitive, perceptible information, tolerance for error, low physical effort, and size and space for approach and use.

This model is used as a guide for the designer in designing different types of the physical environment for different users because it helps to categorize the users according to their characteristics, which are:

- Cognitive abilities: include all complex mental function proses to make an action, for example, decision-making and planning ( ICF illustration library, 2021)
- Social and communication: include all components of the communication process with others by using different devices and methods to deliver or perceive massages ( World Health Organization, 2017)
- Sensory functions: includes touch, smell, visual, and hearing systems ( ICF illustration library, 2021)
- Mobility: the ability to manage body movements such as changing body position or location, carrying objects, and performing physical activities ( ICF illustration library, 2021)

The characteristics of autism are varied in intensity, degree, and amount and manifest differently from person to person and over time. The common characteristics associated with ASD are loosely based on the DSM-5, common features of ASD, and PMM on ASD.

**1.**  Cognitive abilities
**2.**  Social and communication interaction
**3.**  Sensory function
**4.**  Activity performance

There is limited research on how environments may affect behavior and be designed to meet the needs of those with ASD. Also, there is a lack of information on the experience of spaces and perceptions by people with autism. Only two research have been found namely 'MEDIATE – a responsive environment designed for children with autism ( Gumtau *et al.*, 2005) and 'Could light colour and source change mood in children with autism? ( Hernandez Rivera, 2020).

**3- Theoretical underpinnings of design**

Interior designers concentrate on the design of the interior environment with the requirements of the person who will inhabit the space as the driving force behind all design decisions. Human factors, lighting, occupant wellbeing and performance, post-occupancy evaluation, research, theories about the relationship between human behavior and the https://discovery.ucl.ac.uk/id/eprint/10108977/7/Hernandez%20Rivera_10108977_Thesis_redacted.pdf designed environment, and universal design are among the ten knowledge areas covered by the 'Human Environment Needs: Research and Application' (HEN) category.

Experts on ASD consider the first six years of school, from preschool to sixth grade, important in reaching children and laying the groundwork for lifelong learning and general wellbeing. Even when daily activities are meticulously organized, classrooms attended by children with ASD or other children are highly dynamic, unpredictable environments. Because of this instability, examining the architecture of classroom space in schools where children with ASD attend from preschool to sixth grade is difficult. However, the framework identified by ( Guerin, 1992), which recognized the interaction of the human organism (HO), the BTE, the natural environment (NE), and the behavioral environment (BHE).

Autism spectrum disorder (ASD) is a complicated neurological disorder that, until now, has been inscrutable. The population of individuals on the spectrum worldwide is increasing due to the increased awareness. As their numbers grow, professionals in many fields started studying their ASD cases to provide them with a better life ( Hauptman *et al.*, 2019). Individuals on the spectrum are part of a growing population usually ignored in design despite the current tendency to create designs that focus on persons with special needs. There are binding recommendations and laws on designing buildings that respect physical disabilities, and the field is rich in design applications for physical needs ( Sánchez *et al.*, 2011). By contrast, there is utter indifference toward the person with mental health disabilities, even with guidelines for inclusion of children with physical impairment are used and successful, the inclusion of children with mental disabilities lags much behind ( Bilbo *et al.*, 2015) in their research mentioned that "the environment plays a role in human behavior" that greatly influenced the practice of interior architects designing people centers design. ASD children have sensory processing difficulties, which create challenges in understanding the surrounding environment, thus affecting their behaviors negatively ( Sánchez *et al.*, 2011). The built environment can cause extra confusion, which negatively impacts children with ASD due to the challenging developmental disorder of the ASD. Architects and interior architects are responsible for providing an inclusive built environment to improve the quality of life, especially for people with special needs ( Kopec, 2012), yet it is still relatively unnoticed by architects and designers as it's still excluded from building codes or design guidelines. Environmental and behavioral research has profoundly influenced the practice of interior architecture as it's vital to explore the environmental design for autism.

A vast amount of literature has been published on autism in medical and psychological journals over the years. However, few studies from an architectural perspective have been published even though the role of the sensory environment in autistic behavior has been an issue of debate since Leo Kanner first defined the disorder in 1943 ( Kanner, 1943). Recently, architects have become interested in finding out about the relationship between environment and autistic behavior to provide a suitable environment and support wellbeing.

Few interior designers and architects have yet started to define codes and guidelines such as Autism Planning and Design Guidelines 1.0 by Knowlton School of Architecture (2018) as a design solution for ASD to build autism-friendly surroundings that support users with ASD and prepare them to face other environments. The designer's approach usually compares children with ASD and without through their behaviors to find the differences in their needs in the environment ( Delmolino & Harris, 2012). Environmental and behavioral research has profoundly influenced architecture, and there is a growing need and trend toward user-centered and evidence-based design research to create an environment where people with ASD can thrive.

Few scientometric studies have been done to cover the knowledge gap in the ASD research, in that the authors considered examining the topic generally, such as Ozgur & Balci (2022). They found that 'studies on autism have increased significantly in recent years. While approximately 150 studies were published annually in the early 80s, around 6000 studies were published in 2020. In this study, 59653 publications were retrieved, 63.69% of which were journal articles. The remaining publications were reviews, meeting abstracts, editorial materials, proceedings papers, etc. The primary language was English (96.70%) for the retrieved articles. Other languages like Spanish, French, German, Portuguese, Russian, Turkish, etc., were also encountered.

Sweileh *et al.* (2016) studied growth of ASD research from 2005 to 2014 and found a total of 18,490 articles were retrieved. The *Journal of Autism and Developmental Disorders,* with 48,416 citations and an average of 23.59 citations per article, was identified as the most prolific journal. The United States (US) (n = 8594; 46.48 %), the United Kingdom (n = 2430; 13.14 %) and Canada (n = 1077; 5.8 %) have been most productive countries. King's College London (UK) was found on the top of the list for producing literature and receiving citations. 50% of the highly cited articles were in molecular genetics, and the papers with more than 50 citations were published mainly by authors from USA, UK, and Canada.

The above general studies conclude that most literature is based on medical, biotechnology, and psychological perspectives. Most funding agencies are identified as medical institutions, and the US is the most contributing country to generating the literature. Most ASD research in article form and double and triple authorship has more consideration. The citation rate shows an increase in the trend, and the growth in ASD research literature in terms of medical and psychological are noted as a steady increase and are higher in this decade.

However, the development of ASD literature in the architectural field has not been found. Therefore, based on the scientometric analysis, the present study considers estimating and identifying the gaps in the available literature on ASD from the architectural perspective compared to the literature available from the other perspectives, such as medical and psychological.

## Research questions

1) What are the annual research trends and types of ASD research based on architectural design perspectives from 1992–to 2021?
2) Which authors are the most prolific, and what is the authorship trend in autism research?
4) What are the most relevant journals in journals in autism?
5) What are the most important organizations and countries in autism?
6) What are the most used keywords of autism in the field of architecture?
7) What are the most global collaborative countries producing scientific literature on autism?
8) What were the most cited documents and cited references in autism?
9) What are the most influential funding agencies?

## Research methodology

Statistical techniques are used to analyze different types of publications such as books, conferences, journal articles, *etc*., known as bibliometrics. Scientometrics is the sub-field of bibliometrics that studies quantitative means of investigation, scholarly publishing practices, publishing trends, trend topics, *etc*. This study, therefore, applies the scientometric method to ASD in the architecture field to estimate the literature gap. The required literature on autism was retrieved from the Web of Science (as of 4 ^th^ June 2021). The following search query involved in the Web of Science database ( Clarivate Analytics, 2020)

• TOPIC: "autism"
• Refined by: TOPIC: "architecture"
• Further refined by language: English
• Timespan: All years. Indexes: SCI-EXPANDED, SSCI, A&HCI, CPCI-S, CPCI-SSH, ESCI.

**Figure 1.**
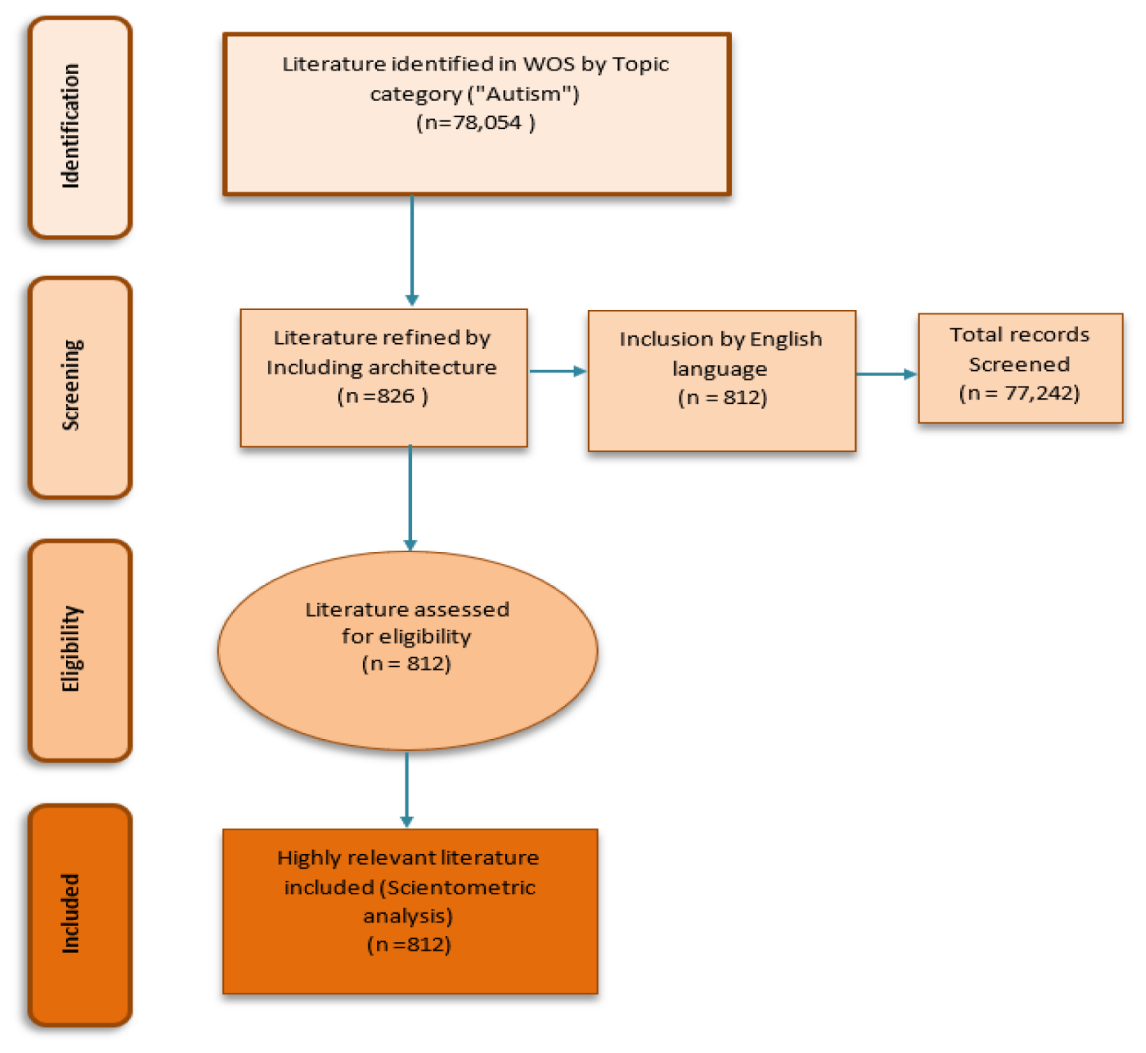
Four phase flow chart of data extraction and filtration process.

812 documents have been retrieved ( Figure 1) for final analysis during 1992–2021. All the research data was downloaded in BibTeX, Tab-Delimited (win), plain text, and analyzed with Microsoft Excel (RRID:SCR_016137; Google Sheets (RRID:SCR_017679) is an open access alternative) and Scientometric and bibliometrics tools, namely Bibexcel ( Persson *et al.*, 2009), Biblioshiny ( Aria & Cuccurullo, 2017), and VOSviewer ( van Eck & Waltman, 2010).

## Results and discussion

From 1992 to 2021, 405 sources were contributed by 5088 authors with 812 papers in autism. Single authored documents were 61 papers; hence authors in autism produce more research in collaboration. The average number of years of publications is 5.74, the average number of citations per document 43.21, and the average number of citations per year per document 5.711. 36,654 references have been consulted to produce 812 research papers. The number of documents per author is 0.16, authors per document are 6.27, Co-authors per document is 8.16, and the collaboration index is 6.71.

### Annual research growth and citation's structure in autism spectrum disorder during 1992–2021

The first research paper on autism was recorded in 1992 with 382 citations (no publication indexed in 1993, 1995, 1996, 1997, and 2003), similar results reported by ( Kumar *et al.*, 2021). Though the research output gradually increases, but shallow up until 2012. The autism research increased markedly after 2013, noticeably more than 50 papers appeared every year after 2013. The year 2019 was the most successful in term of the number of the article (NP=101), followed by the year 2016 and 2017, in which the second highest number of research papers published, coincidently the year 2018 and 2020 have equal number published articles (NP=84) and the year 2021 have 35 papers with 19 citations. The highest number of citations received in 2014 (TC=6634) for 53 publications, followed by the year 2011 (TC=4078) for 31 papers and the year 2010 (TC=3108, TP=34) ( Table 1).

**Table 1.** Annual research growth and citation's structure.

Year	Architecture	TC	Art/Design	NP	TC	Citation sum within h-core	h-index
**1992**	0	0	0	1	382	382	1
**1994**	0	0	0	1	0	0	0
**1998**	0	0	0	2	29	29	2
**1999**	0	0	1	4	235	235	3
**2000**	0	0	0	3	174	174	3
**2001**	0	0	0	1	0	0	0
**2002**	0	0	0	2	126	126	2
**2004**	1	0	0	5	136	136	4
**2005**	0	0	1	3	330	329	2
**2006**	0	0	0	7	557	553	6
**2007**	0	0	0	11	2196	2188	10
**2008**	1	0	1	18	628	614	13
**2009**	2	11	0	22	1547	1522	16
**2010**	0	0	0	34	3108	3000	26
**2011**	0	0	0	31	4078	4008	24
**2012**	0	0	0	31	2360	2301	24
**2013**	1	2	0	51	2467	2239	28
**2014**	0	0	0	53	6634	6305	30
**2015**	2	0	1	58	2093	1818	22
**2016**	3	17	1	85	2549	1972	27
**2017**	1	11	0	85	1965	1396	23
**2018**	1	2	1	84	1726	1240	21
**2019**	3	4	0	101	1451	1035	16
**2020**	1	0	0	84	288	139	10
**2021**	0	0	0	35	19	13	3

*NP=Number of Publications **TC=Total Number of Citations

The authors have scanned all these documents to pinpoint the exact number of research papers purely on architectural design perspective and found a quite low number also, some of it belongs to art and design, these numbers represent the actual gap in the literature, which authors intended to explore and found that gap is quite huge. See ( Table 1).

The first paper on ASD research based on a purely architectural design perspective was published in 2004 and then in 2008. These papers remain unrecognized since they didn't receive a single citation. After a gap of 5 years, another research published under the title "Autism and Architecture" by Segado VF and Segado TA in 2013 received 2 citations; then, in 2015, two research papers were published, again without citations. In 2016 three research papers were published, namely "Interaction Design in the Built Environment: Designing for the Universal User" with 2 citations, "Designed by the pupils, for the pupils: An autism-friendly school" with 7 citations, and "Autism-Friendly Architecture from the outside in and the inside out An explorative study based on autobiographies of autistic people" with 8 citations. In 2017 only one research published under the name "Toward an autism-friendly home environment" by Nagib W and Williams A received 11 citations. A single research in 2018 as, "Sensory Spaces: Sensory Learning - An experimental approach to educating future designers to design autism schools," by Love JS, published in ARCHNET-IJAR, received only 2 citations. Three research papers were published in 2019 under the title Quality of the built environment from the point of view of people with autism spectrum disorder", "The impact of color and light on children with autism in interior spaces from an architectural point of view," and "Studio teaching experiments- spatial transitioning for autism schools" begged 0, 1,1 citations respectively. During pandemic 2020, only one research was published and didn't receive citations, and in 2021 (continuing years) didn't notice any research. Therefore, only 16 ASD research papers were purely related to architectural design from 812 documents noted from 1992 to 2021, with as many as 11 citations. These number of documents and citations reveal that these research areas are not very popular amongst the researchers. Please refer to the recent growth in general ASD research ( Ozgur & Balci, 2022), as mentioned in the literature review.

### Type of research papers

The journal articles (NP=537) were the most preferred form, which agrees with ( Rahaman *et al.*, 2021b). The review found a second preferred form (NP=142), followed by proceedings papers (NP=71) and then meeting abstract (NP=17). Other documents were minor in the list, published only three papers each. On the other hand, the articles also received the highest number of total cations (24922), followed by review (TC=8916) ( Table 2). The research was purely based on an architectural perspective, mostly published as journal articles (13) and then as proceeding papers (3) out of 16. Please refer to ( Table 1) for the total number of pure architectural design research.

**Table 2.** Type of research.

Rank	Document type	NP	TC	Citation sum within h-core	h-index
**1**	Article	538	24922	16725	73
**2**	Review	142	8916	7085	45
**3**	Proceeding’s paper	71	183	92	7
**4**	Meeting abstract	17	0	0	0
**5**	Article Proceedings paper	12	436	419	7
**6**	Review; book chapter	8	383	381	5
**7**	Editorial material	7	162	160	4
**8**	Article; early access	7	11	9	2
**9**	Review; early access	4	1	1	1
**10**	Book chapter	3	55	54	2
**11**	Letter	3	21	21	2

*NP=Number of Publication **TC=Total Number of Citations

### Productive organization

It is evident that the top ten organizational productivity ranges between 25 to 42 publications ( Table 3). The University of Toronto is the leading organization in autism research (NP=42), followed by Vanderbilt University (NP=37), University of California, Los Angeles (NP=35), Yale University (NP=33), and Massachusetts General Hospital (NP=30). Harvard Medical School (NP=25) identified as the minor producer of research in the top ten list. Interestingly, most of the listed organization are in the USA (9 organizations), and one organization from Canada. Stanford University was the most cited organization (TC=6686) for 28 publications, followed by Yale University (TC=6059) for 33 research in autism.

**Table 3.** Top ten organization-wise research in autism.

Rank	Affiliation	Country	NP	TC	Citation sum within h-core	h-index
**1**	Univ Toronto	Canada	42	5358	5162	22
**2**	Vanderbilt Univ	USA	37	5529	5407	27
**3**	Univ Calif Los Angeles	USA	35	5302	5184	23
**4**	Yale Univ	USA	33	6059	5987	22
**5**	Massachusetts Gen Hosp	USA	30	4707	4611	20
**6**	Univ Calif San Francisco	USA	30	4499	4449	17
**7**	Stanford Univ	USA	28	6686	6643	19
**8**	Univ Calif San Diego	USA	27	2541	2476	17
**9**	Hosp Sick Children	Canada	26	4201	4132	18
**10**	Harvard Med Sch	USA	25	1013	950	14

### Productive country

Moreover, it is found that the top eight countries produced over 50 research papers ( Table 4). Only two countries have over 100 articles on autism. The USA had outstanding research output in autism with 433 publications and 27124 citations, followed by the UK (118 publications, 7569 citations), Canada (79 publications, 6816 citations), China (72 publications, 3339 citations), and France (60 publications, 3304 citations). This result parallels the previous scientometric analyses on ASD research, which says that the USA is highly active in producing ASD literature.

The analyses reveal that half of the research in autism contributed by the USA that received the highest number of citations (TC=27124) for 433 publications, followed by the UK with 7569 citations with 118 publications, and Canada with 6816 citations and 79 publications. Australia managed minimum citation (TC=2048) in the list with 46 publications.

**Table 4.** Top ten country-wise research in autism.

Rank	Country	NP	TC	Citation sum within h-core	h-index
**1**	USA	433	27124	19409	76
**2**	UK	118	7569	6612	37
**3**	Canada	79	6816	6281	31
**4**	Peoples R China	72	3339	2970	20
**5**	France	60	3304	3027	22
**6**	Germany	59	5706	5424	24
**7**	Italy	59	3263	2938	21
**8**	Netherlands	55	4490	4213	26
**9**	Australia	46	2048	1856	20
**10**	Sweden	36	4499	4368	20

### The relevant sources in ASD

All the top ten sources have more than 12 publications; coincidentally, six sources ( *American Journal of Human Genetics, American Journal of Medical Genetics Part B-Neuropsychiatric Genetics, Biological Psychiatry, Molecular Autism, Molecular Psychiatry, Neuron*) produced 12 publications each. *Scientific Reports* (Nature Publishing Group) was considered the most relevant source with 14 publications and 203 citations, followed by *Nature Neuroscience* (Nature Publishing Group) with 14 publications and 1986 citations and *Human Molecular Genetics* and *Plos One* with 13 publications each and 1015 and 371 citations, respectively. The analysis reveals that most of the sources belongs to the Q1 category (eight sources), and two in Q2 category. The highest impact factor journal in the list was *Nature Neuroscience* (JIF=20.07), followed by *Neuron* (JIF=14.41) and *Molecular Psychiatry* (JIF=12.38) ( Table 5). These results also revealed the gap in the development of the ASD research literature in terms of architectural design perspective. The top ten journals are again from genetic, molecular biology, and biological psychiatry; this top ten listing lags the source in the areas of architecture or architectural design. Hence, the authors have further explored the sources in which the 16 research papers purely on architectural design have been published. They found very few but popular sources in the field, namely, *Archnet-IJAR International Journal of Architectural Research, International Journal of Arts and Technology, Housing Studies, Journal of Housing and the Built Environment, Journal of Intellectual Disability Research, Journal of Policy and Practice in Intellectual Disabilities, Advances in Human Factors, Sustainable Urban Planning, and Infrastructure.*

**Table 5.** Top ten appropriate sources in autism.

Rank	Source	JIF	Q	Country	Publisher	NP	TC	h_index	g_index	m_index
**1**	*Scientific Reports*	3.99	Q1	UK	Nature	16	203	9	14	1.29
**2**	*Nature Neuroscience*	20.07	Q1	USA	Nature	14	1986	11	14	1.00
**3**	*Human Molecular Genetics*	5.1	Q1	UK	Oxford University Press	13	1015	10	13	0.83
**4**	*Plos One*	2.74	Q2	USA	Public Library of Science	13	371	9	13	0.75
**5**	*American Journal of Human Genetics*	10.5	Q1	USA	Cell Press	12	1093	10	12	0.67
**6**	*American Journal of Medical Genetics* *Part B-Neuropsychiatric Genetics*	3.38	Q2	USA	Wiley-Liss	12	293	8	12	0.67
**7**	*Biological Psychiatry*	12.09	Q1	USA	Elsevier	12	439	10	12	0.77
**8**	*Molecular Autism*	5.86	Q1	USA	BMC	12	226	9	12	0.82
**9**	*Molecular Psychiatry*	12.38	Q1	USA	Nature	12	1290	9	12	
**10**	*Neuron*	14.41	Q1	USA	Cell Press	12	1612	11	12	0.73

*NP=Number of Publication **TC=Total Number of Citations ***JIF=Journal impact factor ****Q=Quartile

### Prolific authors

This analysis reveals that the article range of authors varied between nine and 12. Five authors (Devlin B, Geschwind DH, Scherer SW, State MW, and Wang Y) emerged as the most prolific authors with 13 publications each, 4383, 3409, 3338, 3662, and 333 citations, respectively. Buxbaum JD (Icahn School of Medicine at Mount Sinai) found as the second highest prolific author with 13 publications and 2970 citations, followed by Bourgeron T, Eichler EE, and Li Y with 11 publications and 2142, 1944, and 568 citations, respectively. Casanova MF (University of South Carolina School of Medicine) noted as the least contributed authors in the top ten list with nine publications and 361 citations. Devlin B (Mount Sinai School of Medicine) was the most cited author with 4383 citations for 13 publications, followed by Geschwind DH with 3409 citations for 13 publications, and Wang Y (Carnegie Mellon University) managed only 333 citations for 13 publications. The table also shows that the most prolific authors belong from the USA (7 authors), followed by Canada, France, and China. ( Table 6). It is also revealed that most of the authors belong to medicine and psychology; the authors from the field of architecture are missing from the top 10 list. There are 24 authors found contributing to ASD research in the field of architectural design, amongst them Tufvesson C; Tufvesson J, and Nagib W; Williams A contributing one paper and begged 11 citations, followed by Kinnaer M; Baumers S; Heylighen A (NP=1, TC=8), Mcallister K; Sloan S (NP=1, TC=7). The other authors with one paper received two citations are Segado Vazquez F; Segado Torres A; Dalton C; and Love JS. Shareef SS; Farivarsadri G received one citation for one paper, and the other nine authors didn't receive a citation.

**Table 6.** Top ten most productive authors in autism research.

Rank	Author	Affiliation	Country	NP	TC	h_index	g_index	m_index
**1**	Devlin B	Mount Sinai School of Medicine	USA	13	4383	12	13	0.80
**2**	Geschwind DH	University of California	USA	13	3409	12	13	0.80
**3**	Scherer SW	University of Toronto	Canada	13	3338	12	13	0.80
**4**	State MW	Mount Sinai School of Medicine	USA	13	3662	12	13	0.86
**5**	Wang Y	Carnegie Mellon University	USA	13	333	9	13	1.00
**6**	Buxbaum JD	Icahn School of Medicine at Mount Sinai	USA	12	2970	10	12	0.63
**7**	Bourgeron T	Université de Paris	France	11	2142	9	11	0.60
**8**	Eichler EE	University of Washington,	USA	11	1944	9	11	0.75
**9**	Li Y	Peking University	China	11	568	7	11	1.17
**10**	Casanova MF	University of South Carolina School of Medicine	USA	9	361	9	9	0.45

*NP=Number of Publication **TC=Total Number of Citations

### The pattern of authorship

**Figure 2.**
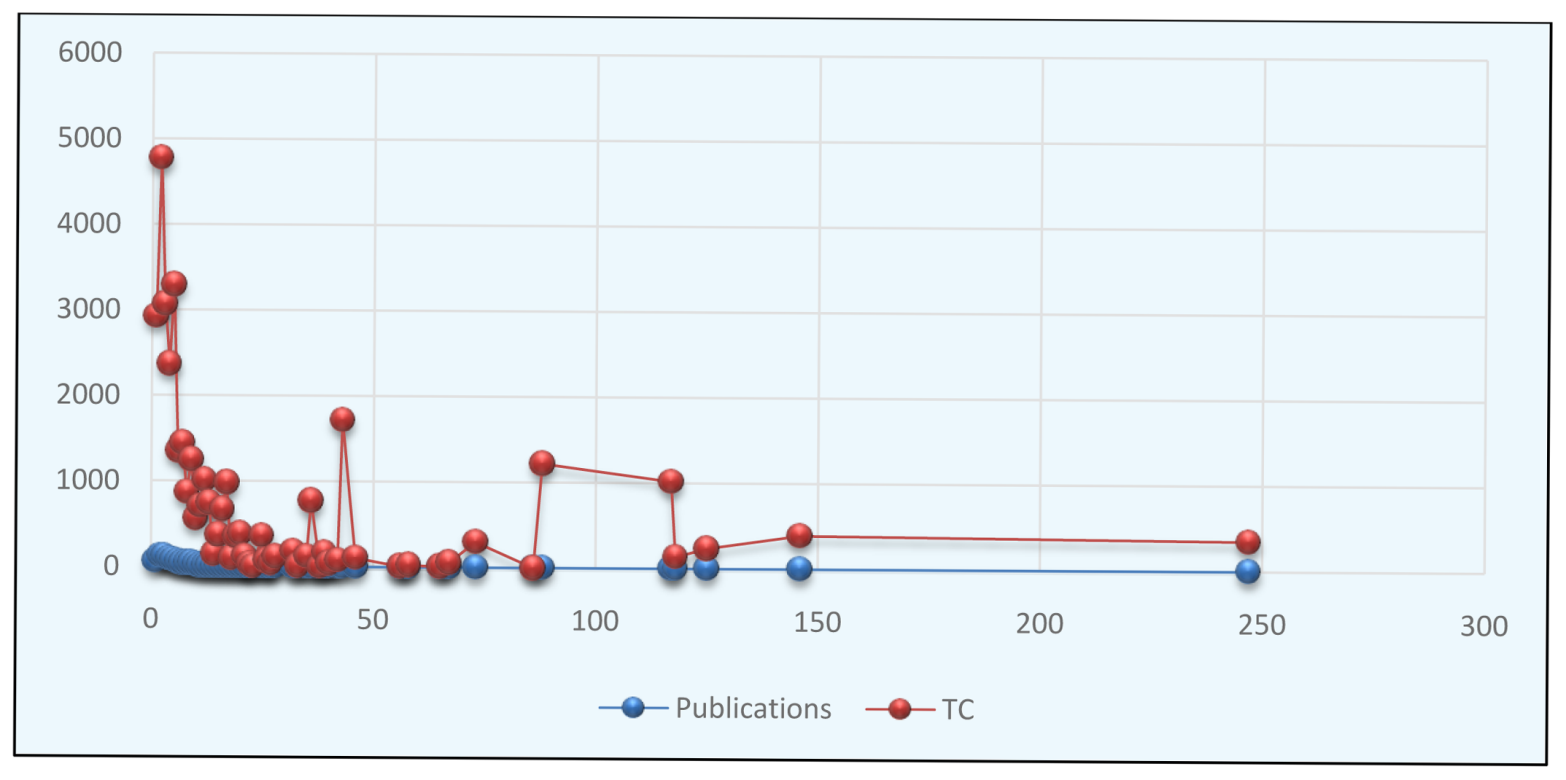
Pattern of authorship in autism.

The Figure 2 illustrated the pattern of authorship in autism literature. It was clear from the figure that the authorship pattern ranged from single to two hundred and forty-seven. The analysis reveals that collaborative research is more prominent among the research of autism over the study period. The top six authorship patterns produced over 50 publications in the field. Three authorship patterns (NP=123) contributed a maximum article in autism, followed by two authorship (NP=120), four authorship (NP=93), five authorship (NP=79), single authorship (NP=61), and six authorship (NP=56). The authorship of 27, 36, 38, 39, 40, 42, 46, 56, 58, 65, 67, 73, 86, 88, 118, 125, 146, and 247 each contributed only single publications in autism. The results also showed that two authorship patterns received the highest number of citations (TC=4775), followed by five authorship (TC=3296) and Three authorship (TC=3071). Rahaman conducted a similar type of authorship pattern analysis ( Rahaman *et al.*, 2021a).

### Mapping co-occurrence of all keywords (author and indexed)

**Figure 3.**
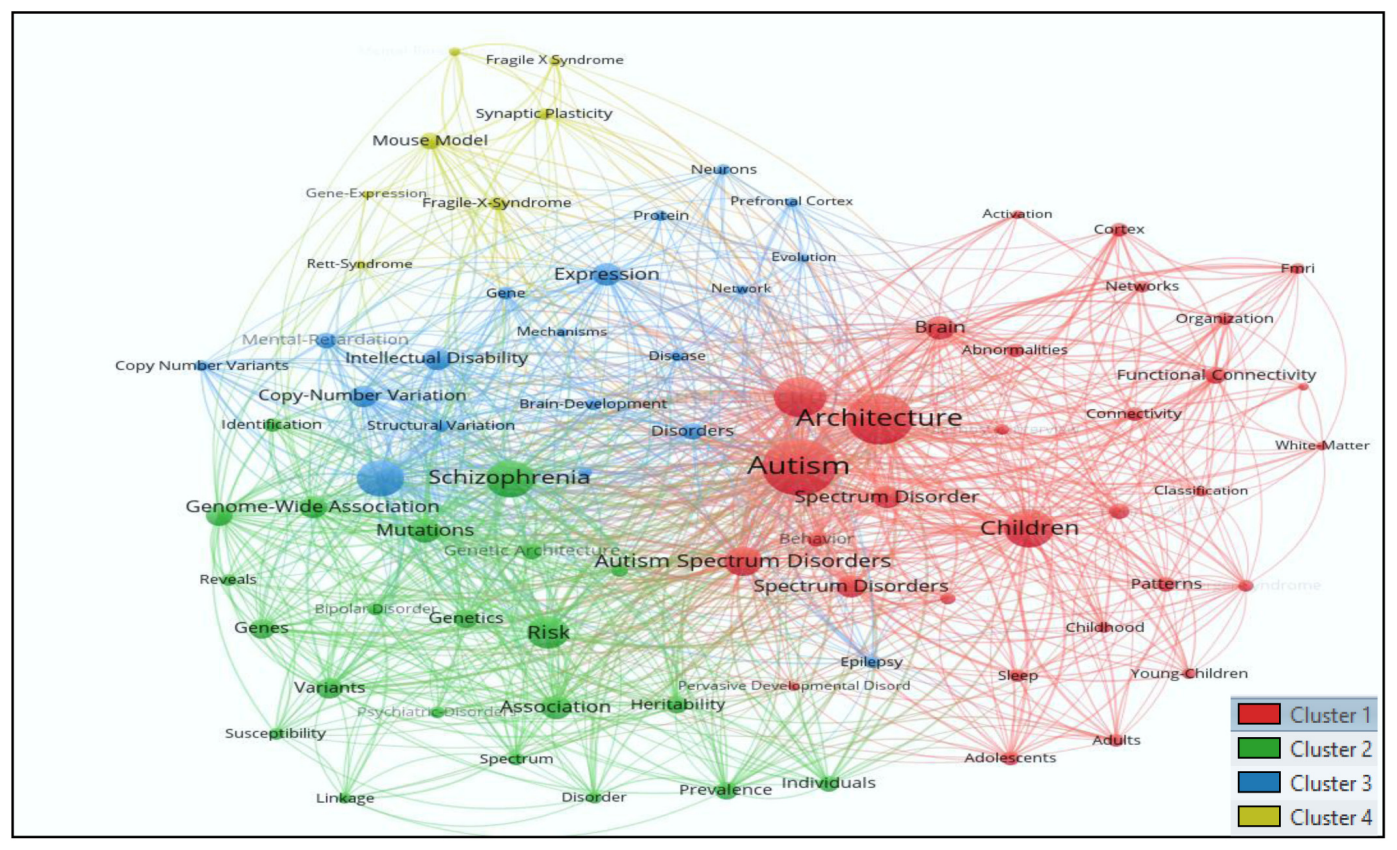
Keyword analysis using Vosviewer.

Figure 3 shows analysis of all keywords used in autism research from 1992–to 2021. The results showed that 3848 keywords appeared in autism research. To map the co-occurrence of all the keywords, minimum of 15 occurrences of keywords were considered for analysis. Out of 3848 keywords, only 79 keywords met the thresholds, and all 79 selected keywords are clustered in Figure 3 with 1737 links and total link strength (5557). The size of the ball indicates a strong network of keywords, with each color representing a distinct cluster.

Cluster *1* comprises 31 keywords (abnormalities, activation, adolescents, adults, architecture Asperger-syndrome, autism, autism spectrum disorder, autism spectrum disorders, behavior, brain, childhood, children, classification, connectivity, cortex, diagnostic interview, fMRI, functional connectivity, high-functioning autism, human cerebral-cortex, meta-analysis, networks, organization, patterns, pervasive developmental disorders, sleep, spectrum disorder, spectrum disorders, white-matter, and young-children).

Cluster *2* has 22 keywords (association, bipolar disorder, copy number variation, disorder, genes, genetic architecture, genetics, genome-wide association, heritability, identification, individuals, linkage, mutations, phenotype, prevalence, psychiatric-disorders, reveals, risk, schizophrenia, spectrum, susceptibility, and variants).

Cluster *3* includes 19 keywords (brain-development, copy number variants, copy-number variation, de-novo mutations, disease, disorders, epilepsy, evolution, expression, gene, intellectual disability, mechanisms, mental-retardation, network, neurodevelopmental disorders, neurons, prefrontal cortex, protein, and structural variation).

Cluster *4* has seven keywords (fragile x syndrome, fragile-x-syndrome, gene-expression, mental-retardation protein, mouse model, rett-syndrome, and synaptic plasticity).

The top ten keywords were autism (frequency=257), architecture (165), autism spectrum disorder (127), children (123), schizophrenia (92), autism spectrum disorders (91), de-novo mutations (86), Risk (73), brain (59) and expression (freq.=55) had weighty number of occurrence with strong total link strength.

Each cluster is based on the theme, which shows the various aspect of the subject and its development. The themes special for architecture or design or built environment are missing to track the development of the subject.

The authors have found a few trendy keywords are missing here, such as acoustics, acoustical control, spatial sequencing, escape spaces, compartmentalization, natural light, fluorescent light, snoezelen, sensory environment, multisensory, neutral sensory, hypersensitive, hyposensitive, sensory trigger, sensory zoning, stimulus level, overstimulating, transition, transition spaces, safety, audio, auditory, auditory processing, distraction, interactive, tactile, tactile sense, altered senses.

### Thematic map by title

**Figure 4.**
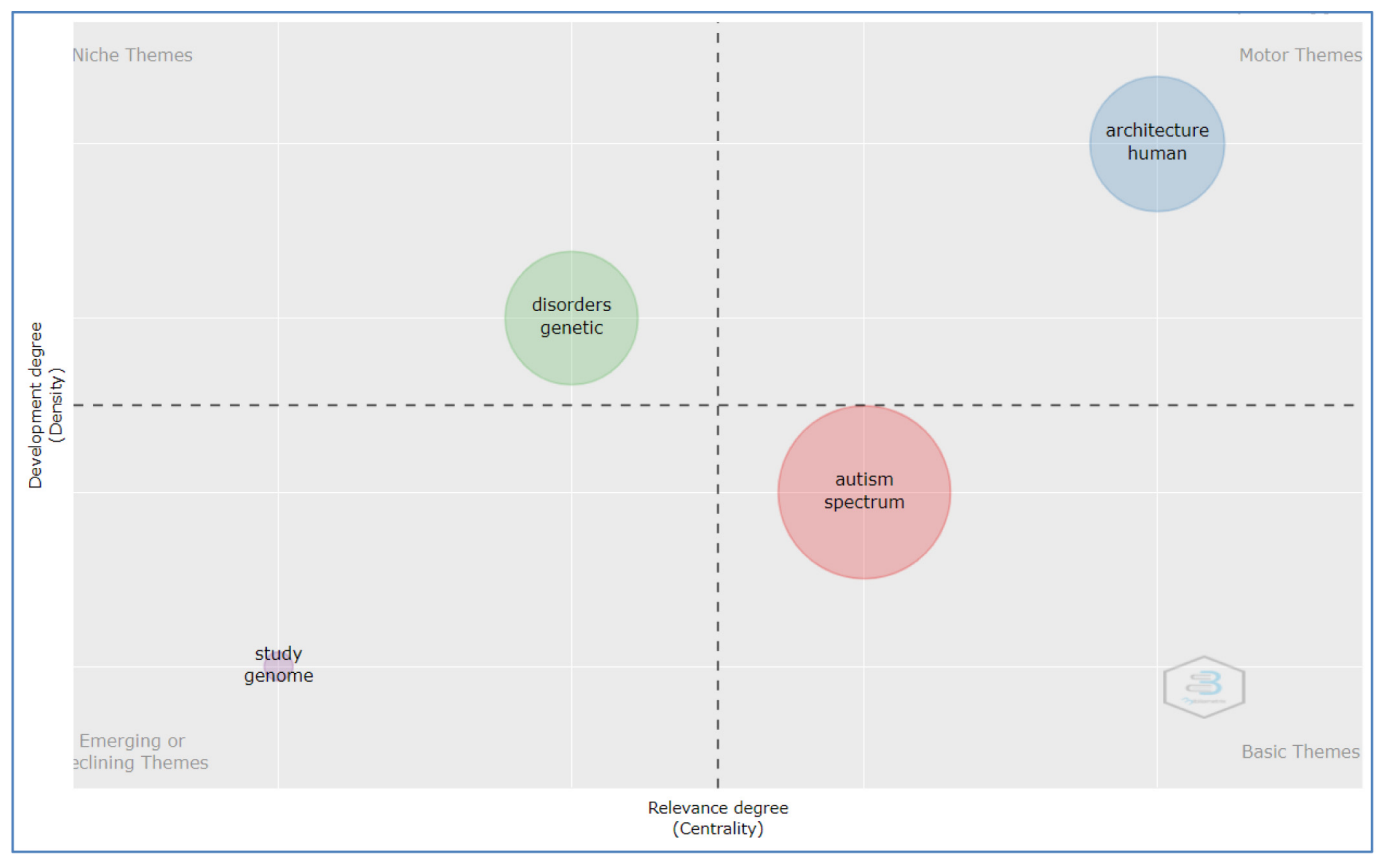
Thematic map by title analysis.

Figure 4 shows four alternative typologies of themes that can be visualized using a thematic map. The thematic parameter is considered the title selected for the field, the minimum number of words selected is 80, and Unigram is selected for the graph.

The basic theme: Autism spectrum which represented by cluster *1* (autism, spectrum, disorder, children, brain, network, functional, connectivity, based, analysis, sleep, neural, developmental, learning, networks, structural, reveals, system, approach, design, matter, review, robot, resting, control, developing and white).

The motor theme: architecture human in cluster *2* (architecture, human, gene, syndrome, social, development, cortical, protein, autistic, model, synaptic, fragile, neuronal, cognitive, ASD, altered, behavior, mental, mice, role, cortex, expression, function, visual, cell, mouse, processing, and activity.

Niche theme: genetic disorder placed in cluster *3* (disorders, genetic, variants, risk, schizophrenia, neurodevelopmental, genes, psychiatric, rare, common, de, genetics, novo, genomic, related, mutations, copy, disease, mechanisms, and sequencing).

Emerging or declining theme: study genome represented by cluster *4* (study, genome, association, wide and evidence).

### Most cited research papers in autism

The top ten papers ( Table 7) have more than 300 citations, published between 2007 and2015. "Large-scale brain networks and psychopathology: a unifying triple network model" (2011) by Menon V, published in *Trends Cogn Sci* was the topmost cited paper (1425 citations) ( Menon, 2011), followed by "Synaptic, transcriptional and chromatin genes disrupted in autism" (2014) by De Rubeis S, appeared in " *Nature*" (1220 citations) ( De Rubeis *et al.*, 2014), "The contribution of de novo coding mutations to autism spectrum disorder" (2014) by Iossifov I, published in *Nature* (1118 citations) ( Iossifov *et al.*, 2014), "Mapping autism risk loci using genetic linkage and chromosomal rearrangements" (2007) by Szatmari (999 citations) ( Szatmari *et al.*, 2007). "Dendritic spine pathology in neuropsychiatric disorders" (2011) by Penzes (838 citations) ( Penzes *et al.*, 2011), and "A genome-wide scan for common alleles affecting risk for autism" was the least cited paper among the top ten (393 citations) ( Anney *et al.*, 2010). It was noticeable that half of the top ten cited papers were published by Nature Publishing Group. The article entitled "Synaptic, transcriptional and chromatin genes disrupted in autism" ( De Rubeis *et al.*, 2014) has the highest total citations per year (152.50).

**Table 7.** Top ten cited papers.

Rank	Title	Author	Yar	Source	TC	TC/Year	N/TC
**1**	Large-scale brain networks and psychopathology: a unifying triple network model ( Menon, 2011)	Menon V	2011	Trends Cogn Sci	1425	129.55	10.83
**2**	Synaptic, transcriptional and chromatin genes disrupted in autism ( De Rubeis *et al*., 2014)	De Rubeis S	2014	Nature	1220	152.50	9.75
**3**	The contribution of de novo coding mutations to autism spectrum disorder ( Iossifov *et al*., 2014)	Iossifov I	2014	Nature	1118	139.75	8.93
**4**	Mapping autism risk loci using genetic linkage and chromosomal rearrangements ( Szatmari *et al*., 2007)	Szatmari P	2007	Nature Genet	999	66.60	5.00
**5**	Dendritic spine pathology in neuropsychiatric disorders ( Penzes *et al*., 2011)	Penzes	2011	Nat Neurosci	838	76.18	6.37
**6**	The autism brain imaging data exchange: towards a large-scale evaluation of the intrinsic brain architecture in autism ( Di Martino *et al*., 2014)	Di Martino A	2014	Mol Psychiatr	769	96.13	6.14
**7**	Insights into Autism Spectrum Disorder Genomic Architecture and Biology from 71 Risk Loci ( Sanders *et al*., 2015)	Sanders Sj	2015	Neuron	563	80.43	15.60
**8**	Most genetic risk for autism resides with common variation ( Gaugler *et al*., 2014)	Gaugler T	2014	Nature Genet	542	67.75	4.33
**9**	Mapping Early Brain Development in Autism ( Courchesne *et al*., 2007)	Courchesne E	2007	Neuron	485	32.33	2.43
**10**	A genome-wide scan for common alleles affecting risk for autism ( Anney *et al*., 2010)	Anney R	2010	Hum Mol Genet	393	32.75	4.30

*N/TC=Normalized total citation

The papers that are well received in architecture or architectural design are not listed here due to a lack of citations than the papers in the other fields; hence, the ASD research in the given fields is less prevalent. The most cited papers in the architectural field are: ' *The building process as a tool towards an all-inclusive school. A Swedish example focusing on children with defined concentration difficulties such as ADHD, Autism, and Down's Syndrome* (2009) and *'Toward an Autism-friendly home environment'* (2017) received 11 citations each. ' *Autism-friendly architecture from the outside in and the inside out: An explorative study based on autobiographies of Autistic people'* (2016) received eight citations, and *'Designed by the pupils, for the pupils: An Autism-friendly school'* (2016) got seven citations.

### Most Cited references in autism research

Table 8 explained the most top ten cited references in autism research. It is clear from the table that all listed references received more than 50 citations. Article entitled "Insights into Autism Spectrum Disorder Genomic Architecture and Biology from 71 Risk Loci" (2015) by Sanders SJ, appeared in 'Neuron' was the most cited (TC=92) reference in autism research ( Sanders *et al.*, 2015), followed by an article named 'Synaptic, transcriptional and chromatin genes disrupted in autism (2014) by De Rubeis S with 91 citations ( De Rubeis *et al.*, 2014), 'and 'The contribution of de novo coding mutations to autism spectrum disorder' (2014) by Lossifov I with 91 citations and appeared in the journal Nature ( Iossifov *et al.*, 2014). The cited references 'De novo gene disruptions in children on the autistic spectrum (2012) by Iossifov I published in 'NEURON' was the most diminutive receiver of citation with 61 TC ( Iossifov *et al.*, 2012). However, the top ten listed references belong to the biotechnology, genetic architecture, and medicinal aspects; the gap identified here is the lack of ASD study on architectural in terms of designer perspective.

**Table 8.** top ten cited references.

Rank	Title	Author	Year	Source	TC
**1**	Insights into Autism Spectrum Disorder Genomic Architecture and Biology from 71 Risk Loci ( Sanders *et al*., 2015)	Sanders SJ	2015	NEURON	92
**2**	Synaptic, transcriptional and chromatin genes disrupted in autism ( De Rubeis *et al*., 2014)	De Rubeis S	2014	Nature	91
**3**	The contribution of de novo coding mutations to autism spectrum disorder ( Iossifov *et al*., 2014)	Iossifov I	2014	Nature	91
**4**	Strong association of de novo copy number mutations with autism ( Sebat *et al*., 2007)	Sebat J	2007	Science	79
**5**	Functional impact of global rare copy number variation in autism spectrum disorders ( Pinto *et al*., 2010)	Pinto D	2010	Nature	77
**6**	De novo mutations revealed by whole-exome sequencing are strongly associated with autism ( Sanders *et al*., 2012)	Sanders SJ	2012	Nature	73
**7**	Sporadic autism exomes reveal a highly interconnected protein network of de novo mutations ( O'Roak *et al*., 2012)	O'roak BJ	2012	Nature	70
**8**	Structural variation of chromosomes in autism spectrum disorder ( Marshall *et al*., 2008)	Marshall CR	2008	AM J HUM GENET	69
**9**	Patterns and rates of exonic de novo mutations in autism spectrum disorders ( Neale *et al*., 2012)	Neale BM	2012	Nature	68
**10**	De novo gene disruptions in children on the autistic spectrum ( Iossifov *et al*., 2012)	Iossifov I	2012	NEURON	61

### Highly influential funding agencies

There are only four funding agencies from the top 10 list which funded more than 100 research papers ( Table 9). National Institutes of Health renowned as leading funding agency (313 publications, 23087 citations), followed by the United States Department of Human Health Services (313 publications, 22759 citations), the National Institute of Mental Health (182 publications, 16164 citations), European Commission (111 publications, 8476 citations), and National Institute of Child Health Human Development (66 publications, 7927 citations). The Wellcome Trust appeared as the least influential funding agency among the top ten (36 publications, 3959 citations). The USA was dominant in the top ten list (six funding agencies), followed by the UK (three funding agencies) and one agency from the EU.

**Table 9.** Top ten funding agencies in autism.

Rank	Funding agencies	Country	NP	% Of 812	TC
**1**	National Institutes of Health	USA	313	38.547	23087
**2**	United States Department of Health Human Services	USA	313	38.547	22759
**3**	National Institute of Mental Health	USA	182	22.414	16164
**4**	European Commission	EU	111	13.67	8476
**5**	National Institute of Child Health Human Development	USA	66	8.128	7927
**6**	National Institute of Neurological Disorders Stroke	USA	62	7.635	7101
**7**	National Institute of General Medical Sciences	USA	50	6.158	2827
**8**	UK Research Innovation	UK	50	6.158	4947
**9**	Medical Research Council UK	UK	48	5.911	5097
**10**	Wellcome Trust	UK	36	4.433	3959

It is to be noted that all funding agencies belong to the health and medicine except one that is the 'UK Research Innovation,' which is a good sign for the researcher belonging to the field of innovation, architecture, design, and creativity to apply for a funded research/projects.

### Country collaboration in autism

**Figure 5.**
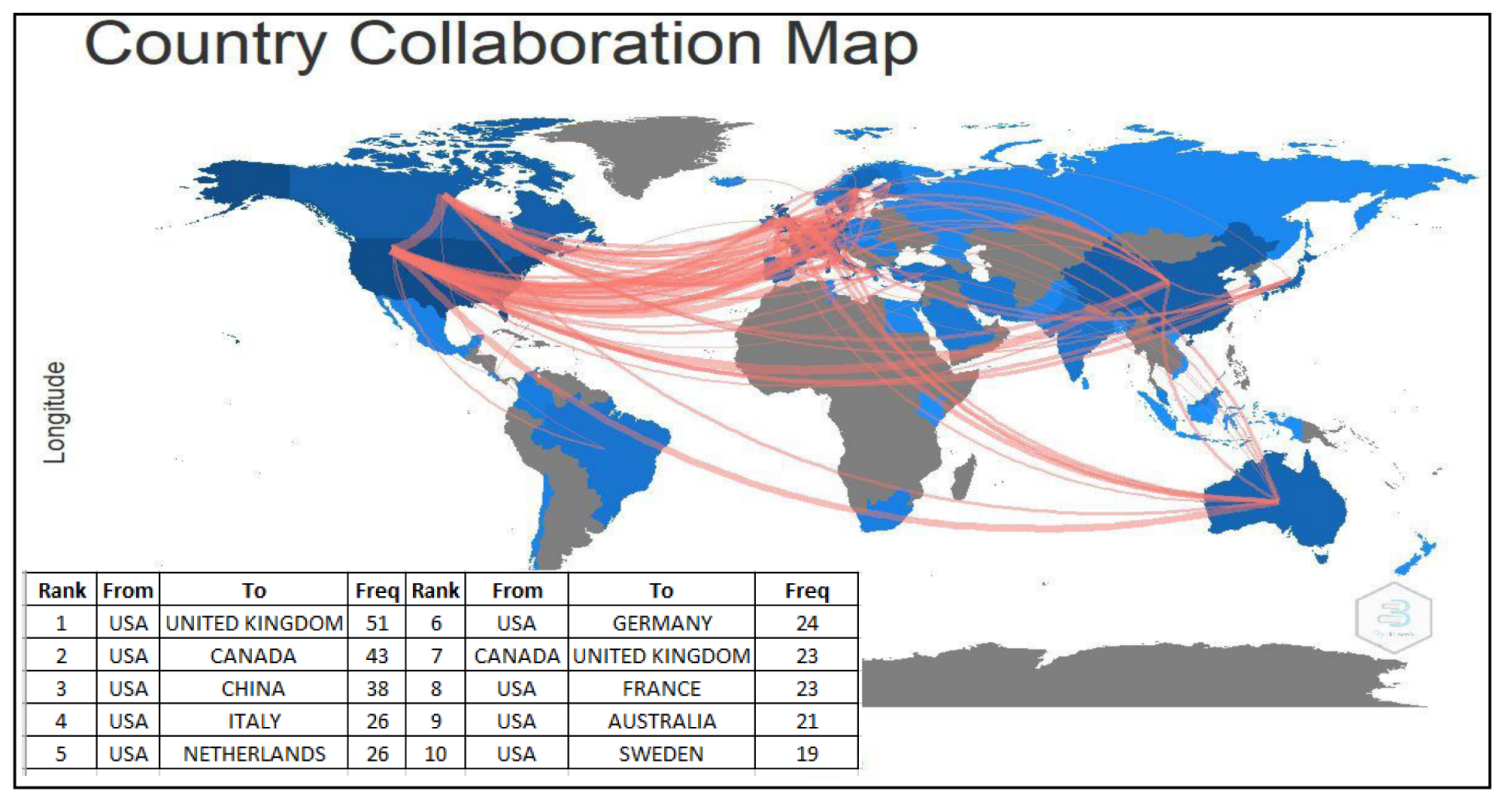
International collaboration map.

The most dominant country collaborations were the USA and United Kingdom (51 publications), followed by the USA and Canada (43 publications), the USA and China (38 publications), the USA and Italy (26 publications), and the USA and the Netherlands (26 publications). The USA with Sweden collaboration (19 publications) was listed at the bottom of the top ten list. It was interesting to show that the USA collaborated with nine countries (the UK, Canada, China, Italy, the Netherlands, Germany, France, Australia, and Sweden). The UK followed this with two countries (the USA and Canada). ( Figure 5).

## Conclusion

This bibliometric study has been proposed to cover the knowledge gap between the amount of literature that has been published on autism in medical and psychological journals over the years and the published research with the architectural and design approach. However, no other bibliometric analysis has been done from 1992 to 2021 that comprehensively evaluates and summarizes the literature, progress, and future directions of this key sub-area of ASD research. The results are eye-opening since only 16 out of 812 papers retrieved are purely relevant to the architectural and designers' perspective. The other papers are medicine, psychology, biotechnology, ICT, computer software design, etc.

The keywords and thematic analyses identified the huge missing gap since all are too generic, therefore, the authors have identified a few missing keywords, which leads them to suggest that more ASD research needs to be done in terms of built environment characteristics, negative sensory experiences, and conducive design features.

The literature review indicated that the performance prediction model (PPM) needs more research since, for over 2 decades, only 2 projects (cited in literature review) focused on describing the transactions between the users and their physical environment through the behavior. It also suggested that designers need to work more in defining codes and guideline to build autism-friendly environment to support people with ASD. The top ten analyses of the country, institution and funding agencies show that the USA is highly active in producing ASD research. Stanford University is noted as the most cited organization might be due to its own program for Autism research, extending a good platform for the researchers in this field. The 'UK Research Innovation' is the only funding agency to provide opportunities to researchers in design and innovation.This research also leads researchers to discover the most influential publications, authors, and journals in this field.

Here are a few noteworthy emerging trends (the missing gap in this study) in ASD research where researchers in the field of architectural design and built environment can dwell in are; *acoustical control, spatial sequencing, escape spaces, compartmentalization, snoezelen, sensory environment, sensory zoning, overstimulation, transition spaces, safety, auditory processing, tactile sense, altered senses*.

## Data availability

### Underlying data

Zenodo: Underlying data for 'autism spectrum disorder in architecture perspective: A review of the literature and bibliometric assessment of research indexed in Web of Science'. https://doi.org/10.5281/zenodo.5080242

Data are available under the terms of the Creative Commons Attribution 4.0 International license (CC-BY 4.0).
