# Supplementary material for: Autism spectrum disorder in architecture perspective: a review of the literature and bibliometric assessment of research indexed in Web of Science
Source: F1000Res. 2022 Jul 25;10:1087. Originally published 2021 Oct 26. [Version 2] doi: 10.12688/f1000research.54437.2 (PMC9326241; doi:10.12688/f1000research.54437.2)
Supplement: Peer review history

# Peer review: Reviewer response for version 2

**DOI:** 10.5256/f1000research.135006.r145220

**Published:** 2022-07-26

**Authors:** Peter Kokol

**Affiliations:** 1 Faculty of Electrical Engineering and Computer Science, University of Maribor, Maribor, Slovenia

Authors answered to my comments, and I would like to approve the article.

Is the work clearly and accurately presented and does it cite the current literature?

Partly

If applicable, is the statistical analysis and its interpretation appropriate?

Not applicable

Are all the source data underlying the results available to ensure full reproducibility?

Yes

Is the study design appropriate and is the work technically sound?

Partly

Are the conclusions drawn adequately supported by the results?

Partly

Are sufficient details of methods and analysis provided to allow replication by others?

Partly

Reviewer Expertise:

NA

I confirm that I have read this submission and believe that I have an appropriate level of expertise to confirm that it is of an acceptable scientific standard.

# Peer review: Reviewer response for version 1

**DOI:** 10.5256/f1000research.57922.r101414

**Published:** 2021-12-15

**Authors:** Hashem Hussein Al-Attas

**Affiliations:** 1 Deanship of Library Affairs, King Fahd University of Petroleum and Minerals, Dhahran, Saudi Arabia

The authors have collected an exclusive dataset from Web of Science using quantitative methodology. The bibliometric method to map the global research publication on autism spectrum disorder in architecture perspective, definitely contributes to the field and other researchers. It can help them decide the most productive country, journals, organization, pattern of authorship, most important author keywords, research themes, and new international collaboration. The analyses, results, and interpretation display interesting and beneficial data. Moreover, quality of the text is good. There are a few unnecessary capitalizations in the sentences, but ignorable. Overall the paper represents valuable information regarding autism spectrum disorder in architecture research.

Is the work clearly and accurately presented and does it cite the current literature?

Yes

If applicable, is the statistical analysis and its interpretation appropriate?

Yes

Are all the source data underlying the results available to ensure full reproducibility?

Yes

Is the study design appropriate and is the work technically sound?

Yes

Are the conclusions drawn adequately supported by the results?

Yes

Are sufficient details of methods and analysis provided to allow replication by others?

Yes

Reviewer Expertise:

I have enough knowledge in the field of bibliometric and scientometric studies.

I confirm that I have read this submission and believe that I have an appropriate level of expertise to confirm that it is of an acceptable scientific standard.

# Peer review: Reviewer response for version 1

**DOI:** 10.5256/f1000research.57922.r97972

**Published:** 2021-11-01

**Authors:** Peter Kokol

**Affiliations:** 1 Faculty of Electrical Engineering and Computer Science, University of Maribor, Maribor, Slovenia

The authors performed an interesting bibliometric study. They focused mainly on quantitative aspects of the research on autism-related architectural design. However, the paper should be amended in some aspects to make it more informative for readers and to make the study repeatable.

 First, the introduction and the literature review should be extended with a description of bibliometrics, evidence of its successful use (and the reason why they selected bibliometrics as a knowledge synthesis method), the bibliometrics tools used should be shortly described and their use in the study stated more clearly. There are already some bibliometrics studies on autism and other disabilities already published, authors should point to them in the literature review and connect their research to already performed studies (they should also compare their results to results of similar studies in the discussion section).

 In the results section, they should point out which bibliometric tool was used to produce them. The results should also be extended with qualitative aspects, actually, the discussion is mostly missing. What is the meaning of the results, who can use them, and for what purpose. In the conclusion, authors mention that research gaps, research directions could be derived from their results, but the readers could benefit much more if the authors themselves will reveal hot topics, gaps, directions, etc. Authors should describe revealed clusters from keywords analysis in more detail. They should use thematic or content analysis to name and describe clusters, point to relevant literature, etc.

Is the work clearly and accurately presented and does it cite the current literature?

Partly

If applicable, is the statistical analysis and its interpretation appropriate?

Not applicable

Are all the source data underlying the results available to ensure full reproducibility?

Yes

Is the study design appropriate and is the work technically sound?

Partly

Are the conclusions drawn adequately supported by the results?

Partly

Are sufficient details of methods and analysis provided to allow replication by others?

Partly

Reviewer Expertise:

Computer science, bibliometrics, machine learning, health informatics

I confirm that I have read this submission and believe that I have an appropriate level of expertise to state that I do not consider it to be of an acceptable scientific standard, for reasons outlined above.

# Response

**Published:** 2022-06-10

**Authors:** Deema Al-Saleh

**Affiliations:** Imam Abdulrahman bin Faisal University , Saudi Arabia

- Comment of reviewer: The authors performed an interesting bibliometric study. They focused mainly on quantitative aspects of the research on autism-related architectural design. However, the paper should be amended in some aspects to make it more informative for readers and to make the study repeatable.

**Authors Response**: Done, the authors have worked more on the dataset and amended the annual literature growth table 1 to compare the literature growth in general with literature growth from the architectural and designers' perspective.

- Comment of reviewer: First, the introduction and the literature review should be extended with a description of bibliometrics, evidence of its successful use (and the reason why they selected bibliometrics as a knowledge synthesis method), the bibliometrics tools used should be shortly described and their use in the study stated more clearly. There are already some bibliometrics studies on autism and other disabilities already published, authors should point to them in the literature review and connect their research to already performed studies (they should also compare their results to results of similar studies in the discussion section).

**Authors Response**: Done, the introduction and the literature review have been extended and previous bibliometric studies included and compared the results.

- In the results section, they should point out which bibliometric tool was used to produce them. The results should also be extended with qualitative aspects, actually, the discussion is mostly missing. What is the meaning of the results, who can use them, and for what purpose. In the conclusion, authors mention that research gaps, research directions could be derived from their results, but the readers could benefit much more if the authors themselves will reveal hot topics, gaps, directions, etc. Authors should describe revealed clusters from keywords analysis in more detail. They should use thematic or content analysis to name and describe clusters, point to relevant literature, etc.

**Authors Response**: Done, all the issues raised have been addressed.
